# Gain-of-function and loss-of-function *GABRB3* variants lead to distinct clinical phenotypes in patients with developmental and epileptic encephalopathies

**DOI:** 10.1038/s41467-022-29280-x

**Published:** 2022-04-05

**Authors:** Nathan L. Absalom, Vivian W. Y. Liao, Katrine M. H. Johannesen, Elena Gardella, Julia Jacobs, Gaetan Lesca, Zeynep Gokce-Samar, Alexis Arzimanoglou, Shimriet Zeidler, Pasquale Striano, Pierre Meyer, Ira Benkel-Herrenbrueck, Inger-Lise Mero, Jutta Rummel, Mary Chebib, Rikke S. Møller, Philip K. Ahring

**Affiliations:** 1 Brain and Mind Centre, School of Pharmacy, Faculty of Medicine and Health, The University of Sydney, Sydney, New South Wales Australia; 2 Department of Epilepsy Genetics and Personalized Treatment, Member of the ERN EpiCARE, The Danish Epilepsy Centre, Dianalund, Denmark; 3 Department of Regional Health Research, University of Southern Denmark, Odense, Denmark; 4 Department of Neuropediatrics and Muscle Disorders, Medical Center-University of Freiburg, Freiburg, Germany; 5 Department of Paediatrics and Department of Neuroscience, Cumming School of Medicine, University of Calgary, Calgary, AB Canada; 6 Hotchkiss Brain Institute and Alberta Children’s Hospital Research Institute, University of Calgary, Calgary, AB Canada; 7 Department of Medical Genetics, Member of the ERN EpiCARE, University Hospitals of Lyon (HCL), Lyon, France; 8 Institut Neuromyogène, CNRS UMR 5310 - INSERM U1217, Université de Lyon, Université Claude Bernard Lyon 1, Lyon, France; 9 Department of Paediatric Clinical Epileptology, Sleep Disorders and Functional Neurology, Member of the ERN EpiCARE, University Hospitals of Lyon (HCL), Lyon, France; 10 Department of Clinical Genetics, Erasmus MC, Rotterdam, The Netherlands; 11 IRCCS Institute “Giannina Gaslini”, Genova, Italy; 12 Department of Neurosciences, Rehabilitation, Ophthalmology, Genetics, Maternal and Child Health, University of Genova, Genova, Italy; 13 Pediatric Neurology Department, Phymedexp, Montpellier University, Inserm, CRNS, Montpellier University Hospital, Montpellier, France; 14 Sana-Krankenhaus Düsseldorf-Gerresheim, Academic Teaching Hospital der Heinrich-Heine-University Düsseldorf, Düsseldorf, Germany; 15 Department of Medical Genetics, Oslo University Hospital, Oslo, Norway; 16 Department of Neurohabilitation, Oslo University Hospital, Oslo, Norway; 17 Present Address: School of Science, Western Sydney University, Sydney, NSW Australia

**Keywords:** Epilepsy, Encephalopathy

## Abstract

Many patients with developmental and epileptic encephalopathies present with variants in genes coding for GABA_A_ receptors. These variants are presumed to cause loss-of-function receptors leading to reduced neuronal GABAergic activity. Yet, patients with GABA_A_ receptor variants have diverse clinical phenotypes and many are refractory to treatment despite the availability of drugs that enhance GABAergic activity. Here we show that 44 pathogenic *GABRB3* missense variants segregate into gain-of-function and loss-of-function groups and respective patients display distinct clinical phenotypes. The gain-of-function cohort (*n* = 27 patients) presented with a younger age of seizure onset, higher risk of severe intellectual disability, focal seizures at onset, hypotonia, and lower likelihood of seizure freedom in response to treatment. Febrile seizures at onset are exclusive to the loss-of-function cohort (*n* = 47 patients). Overall, patients with *GABRB3* variants that increase GABAergic activity have more severe developmental and epileptic encephalopathies. This paradoxical finding challenges our current understanding of the GABAergic system in epilepsy and how patients should be treated.

## Abstract (web summary)

Genetic variants of γ-aminobutyric acid (GABA_A_) receptors are associated with early onset epilepsies. Here, the authors show that functional loss or gain-of-function defines clinical outcomes, with gain-of-function variants unexpectedly more severe.

## Introduction

Developmental and Epileptic Encephalopathies (DEEs) are severe neurological conditions that occur in infancy and are associated with epilepsy and intellectual disability (ID). Genetic factors play a major role and within recent years, a plethora of genetic variants in genes that encode γ-aminobutyric acid type A (GABA_A_) receptor subunits have been identified in patients with DEE^1^. Accordingly, the GABAergic system plays a ubiquitous role in epilepsy aetiology and drugs that enhance GABAergic activity are commonly used anti-seizure medications (ASM).

The *GABRB3* gene is one of 19 genes that code for a GABA_A_ receptor subunit. It has one of the highest prevalence of pathogenic variants^1^, and studies indicate that in these patients, epilepsy arises from either a disruption in receptor expression or a loss of receptor function that results in decreased GABAergic activity^2^. Reduced GABAergic inhibition then ultimately causes over-excitation in the brain circuitry leading to epilepsy^2,3^. This simple, generalist explanation does not, however, explain why a single, monogenic cause of disease leads to a wide spectrum of clinical phenotypes in patients with variants in the *GABRB3* gene^4^. Neither does it explain why many patients are refractive to ASMs that enhance GABAergic signalling^5^.

In this study, we address these two conundrums: Firstly, we interrogated the GABAergic function of epilepsy-associated *GABRB3* variants from a total of 85 patients. Secondly, we performed a comprehensive genotype/phenotype correlation of clinical presentations against the functional outcomes. Contrary to previous reports of epilepsy exclusively caused by variants with a loss of GABA inhibition, we discovered that approximately half of the missense variants in this study enhanced GABAergic activity (gain-of-function), while the remaining half were loss-of-function variants. These two types of molecular phenotypes strongly correlated with two distinct clinical populations differing in age of seizure onset, seizure type, intellectual impairment, and treatment response. Hence, we show that *GABRB3*-mediated DEEs essentially encompass two distinct groups of patients, those with a gain-of-function and those with a loss-of-function variant. This finding provides an explanation for how GABA_A_ receptor missense variants can lead to such a remarkably diverse set of clinical manifestations.

The combination of phenotypic, genetic, and functional studies has resulted in remarkable advances in the field of epilepsy genetics. This has enabled the understanding of molecular and cellular disease mechanisms and a steadily increasing number of tailored treatments. Examples of this exist for genetic variants that cause altered ion channel activity in genes including *SCN1A*, *SCN2A*, *SCN8A*, *KCNA2* and *KCNQ2* which, like our findings, can frequently be grouped into loss-of-function or gain-of-function mechanisms^6–11^. Importantly, the variant category not only corresponded to clinical disease outcomes, but also to differences in drug response. With a general trend towards targeted treatment in the genetic epilepsies, the ability to distinguish between loss-of-function and gain-of-function variants, may also turn out to be clinically relevant for *GABRB3* variants in the future. To assist clinicians in identifying the functional category of *GABRB3* variants, we therefore developed a predictive tool that can be utilized to determine whether patients may have a gain- or a loss-of-function GABA_A_ receptor variant.

## Results

**Fig. 1:**
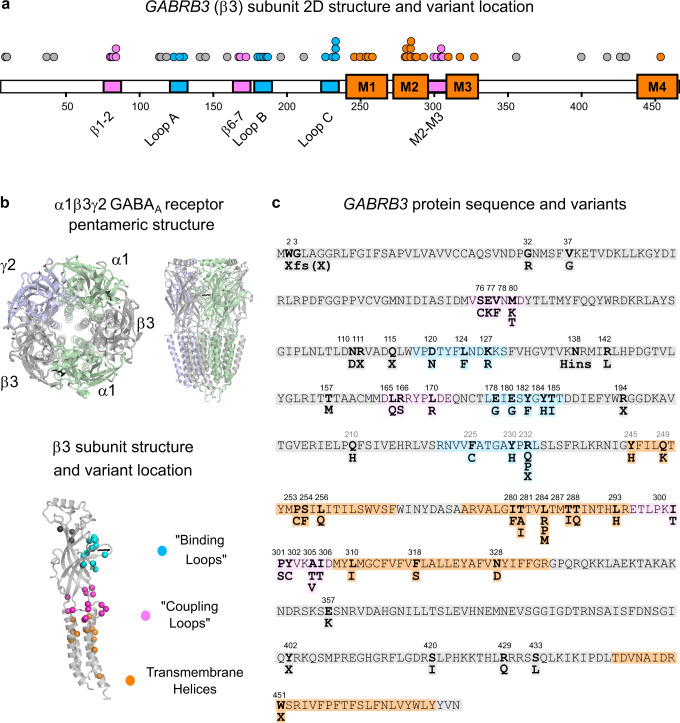
*GABRB3* epilepsy-associated variants. **a** Two-dimensional representation (top) of a GABA_A_ receptor β3 subunit (pdb:6hup)^15^ highlighting binding loops (blue), coupling loops (pink) and four transmembrane regions (orange). Dots represent individual variants associated with DEE. **b** 3D cartoon structure of the pentameric α1β3γ2 GABA_A_ receptor from above and the side. α1 (pale green) β3 (silver) and γ2 (light purple) subunits are colour coded and the GABA molecule is represented as black sticks. A 3D cartoon structure of a β3 subunit showing the location of variants as spheres coloured by their location in the binding loops (blue), coupling loops (pink), transmembrane regions (orange) or elsewhere (dark grey). **c** The *GABRB3* sequence is shown with missense amino acid variants numbered and in bold. Structural features of the binding, coupling loops and transmembrane regions are coloured.

We identified 54 epilepsy-associated missense variants within the coding region of *GABRB3* (Fig. 1a, b). In addition, a further nine truncating, frameshift or deletion variants were found scattered along the β3 subunit polypeptide (Fig. 1c). Based on the American College of Medical Genetics and Genomics (ACMG) criteria, all variants apart from two found in the gnomAD database were classified as pathogenic or likely pathogenic, for de novo and inherited variants, respectively, on the criteria of null variants in a gene where loss-of-function is a known mechanism of epilepsy^12^. A total of 85 individual patients harboured these variants, including 67 with de novo and 15 with familial variants (Supplementary Table S1). We collated the variant identity and all critical clinical features including age-of-onset, seizure types and severity of intellectual disability amongst others.

**Fig. 2:**
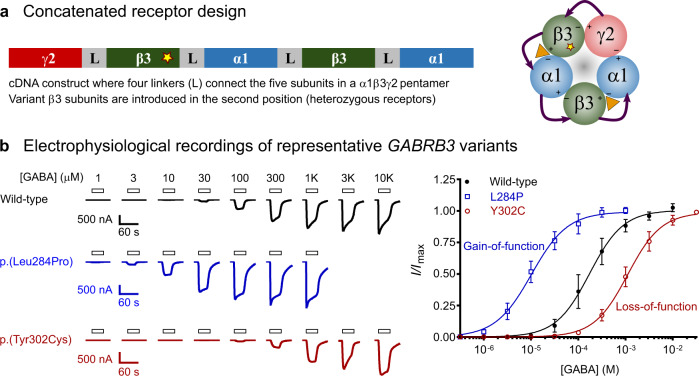
Functional evaluation of *GABRB3* missense variants. **a** Concatenated receptor design showing the γ2-β3-α1-β3-α1 DNA construct used to introduce single missense variants. A pentameric receptor that arranges with a single β3 variant (star) is depicted. **b** Raw electrophysiological recordings and concentration–response curves at selected concatenated γ2-β3-α1-β3-α1 wild-type (black), p.(Leu284Pro) (blue) and p.(Tyr302Cys) (red) β3 variant receptors that represent a gain-of-function and a loss-of-function variant, respectively. Bars indicate GABA applications and scale bars for each trace are shown. Peak current amplitudes were measured at each GABA concentration and a Hill equation was fitted to the data. Thereafter the dataset was normalized to its respective maximum current amplitude for each cell. Black circles (wild-type, *n* = 12 independent cells), blue open squares (p.(Leu284Pro), *n* = 12) and red open circles (p.(Tyr302Cys), *n* = 12) represent the mean ± s.d (*n* ≥ 12) and the lines represent the fitted Hill equation at each receptor. Gain-of-function variants shift the concentration–response curve to the left, loss-of-function to the right. Source data are provided in the Source Data file.

The spectrum of epilepsy syndromes was strikingly diverse in patients with *GABRB3* variants (Supplementary Table S1), showing a wide range of age of seizure onset between 0 and 14 years of age and different seizure types. To address whether different functional changes in GABA_A_ receptor activity are responsible for the observed range of clinical outcomes, we performed a systematic, unbiased functional evaluation of the 54 *GABRB3* variant. As heterozygous receptors are expected to constitute 50 % of expressed receptors in patients, we utilized an expression technique of concatenated receptors for assessing the function of variants (Fig. 2a, Supplementary Fig S1). This methodology guarantees the expression of uniform populations of heterozygous receptors. Furthermore, a range of steps were taken to ensure that highly robust data were obtained from these experiments, which enables reclassification of variants according to the ACMG criteria (see methods).

### Functional analysis of *GABRB3* variants

**Fig. 3:**
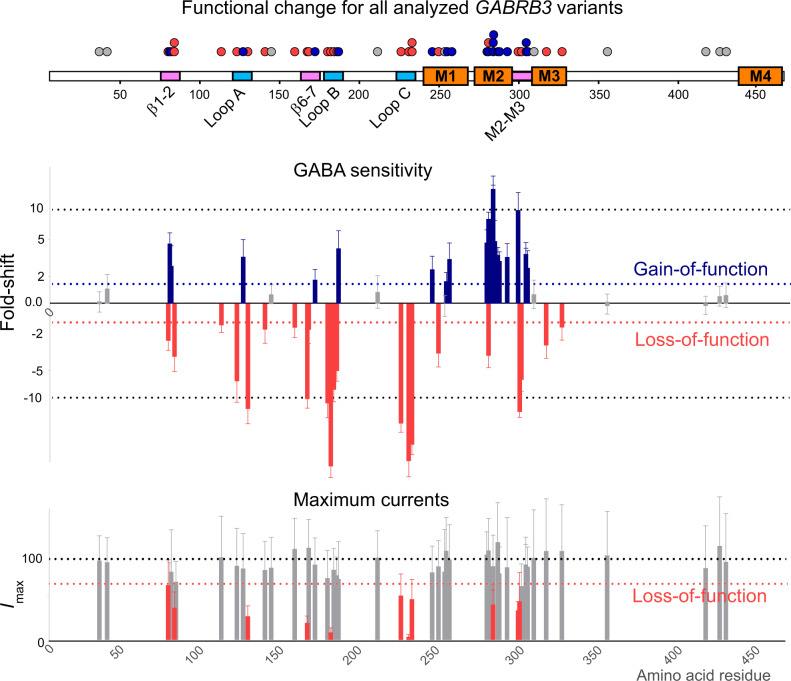
Changes in GABA sensitivity and maximum currents of *GABRB3* missense variants. A two-dimensional representation (top) of a GABA_A_ receptor β3 subunit depicting binding loops (blue), coupling loops (pink) and four transmembrane regions (orange) is shown. Bar graph summarizing the fold-shift in GABA sensitivity (mean ΔlogEC_50_ ± s.d.) and maximum current (mean *I*_max_ ± s.d.) for each variant according to their amino acid residue. At GABA sensitivity, blue bars indicate variants that have significantly increased the GABA sensitivity with the direction of the bars upwards, red bars indicate variants that have significantly decreased the GABA sensitivity with the direction of the bars downwards and grey bars indicate no significant change. Significance was determined by one-way ANOVA (*F* (53, 725) = 355.5) and multiple comparisons with corrected Dunnett’s post-hoc test (*p* < 0.0001, *n* = 135 independent cells (wild-type); 10 (G32R, M80T, R142L, A305T, A305V, I306T); 11 (E77K, M80K, D120N, T157M, Y182F, Y184H, Q249K, S254F, L256Q, T281I, L284M, T287I, T288N, L293H, P301L); 12 (S76C, V78F, L124F, L170R, E180G, F225C, Y230H, Y245H, P253L, I280F, L284R, L284P, Y302C, F318S, N328D, E357K, S420I, R429Q, S433L); 13 (E178G, T185I); 14 (K127R, L165Q, Q210H, R232Q, T281A); 15 (V37G, N110D, ins138_139H, R166S, I300T); 17 (L310I). At maximum current, grey bars indicate no significant change and red bars indicate significantly reduced maximum currents. Significance was determined by one-way Kruskal–Willis ANOVA (H(55, 1653) = 674.4) and multiple comparisons with corrected Dunn’s post-hoc test (*p* < 0.0001, *n* = 256 independent cells (wild-type); 22 (R232P, L284R, I300T, Y302); 23 (D120N, L124F, L284, P301L, A305T); 24 (E77K, V78F, M80K, K127R, L165Q, Q210H, R232Q, Y245H, P253L, S254F, L256Q, I280F, T281A, T281I, L284M, T287I, T288N, L293H, A305V, I306T); 26 (M80T, Y184H, Q249K); 27 (G32R, S76C, R142L, L170R, E180G, Y182F); 28 (L310I); 29 (F318S, E357K, R429Q, S433L); 30 (ins138_139H, Q210H); 33 (V37G), p.(R166S); 36 (S420Ile); 39 (N328D). Individual data points are in the supplementary information (Supplementary Fig S2). Source data are provided in the Source Data file.

The sensitivity of receptors to GABA is a key, fundamental property of GABA_A_ receptors. Hence the receptors sensitivity to GABA (GABA EC_50_ values) was determined for 54 missense variants and compared to wild-type receptors (Fig. 2b, c). Of the 54 variants studied, 20 variants had between a 1.3 and 22-fold reduction in the GABA EC_50_ value (*p* < 0.0001, one-way ANOVA, *F* (53, 725) = 355.5, Dunnett’s post-hoc test) (Fig. 3, Supplementary Fig S2 and Table S2). A reduction in GABA EC_50_ infers that the receptors are more sensitive to GABA and hence should be denoted as having a gain-of-function (GOF) trait. Of the remaining variants, 24 variants had between 1.5 and 54-fold increase in the GABA EC_50_ value (*p* < 0.0001, one-way ANOVA, Dunnett’s post-hoc test) (Fig. 3). An increase in the GABA EC_50_ infers that the receptor is less sensitive to GABA and should be denoted as having a loss-of-function (LOF) trait. The remaining ten variants did not significantly alter GABA sensitivity as demonstrated by no significant change in GABA EC_50_ value.

Another important property of GABA_A_ receptors in a cell, is the amount of current that flows through the channel as measured by the maximum GABA-activated current amplitude. A reduction in maximum GABA current amplitudes could infer reduced trafficking of receptors to the cell surface or reduced ability of GABA to gate the receptor (*de facto* haploinsufficiency) and therefore might reveal further LOF variants. Of the 54 variants evaluated, the ten variants with unchanged GABA sensitivity also showed no significant change in maximal current amplitudes indicating that these variants do not have clear LOF or GOF traits (Fig. 3). Of the 20 variants observed to have GOF GABA sensitivity, only three variants showed a significant reduction in maximum GABA-activated current amplitudes, however, these three variants also caused spontaneous receptor activity (currents in the absence of GABA) which leads to underestimation in the current measurements (Supplementary Fig S3). Finally, of the 24 variants with LOF in GABA sensitivity, ten also displayed a significant reduction in maximum currents amplitude (*p* < 0.0001, one-way ANOVA H(55, 1653) = 674.4, Dunn’s post-hoc test). Thus, maximal GABA current measurements did not reveal any additional LOF variants.

In summary, functional analysis of *GABRB3* epilepsy-associated variants, as measured by GABA sensitivity and maximum GABA current amplitudes, clearly demonstrated that two distinct functional categories of GABA_A_ receptors were identified: an unexpected GOF receptor characterized by an increase in GABA sensitivity without significant reduction in maximum GABA amplitude and the classical LOF receptor, characterized by a decrease in GABA sensitivity with or without a reduction in maximum GABA amplitude. Remarkably, different variants at the same amino acid residue can cause opposite functional outcomes. This is exemplified by two variants in the Thr281 residue where the p.(Thr281Ala) variant caused GOF and p.(Thr281Ile) caused LOF. Extended analysis of the properties of all variants are available in the supplementary information (Supplementary Figs. S4–7).

Following this analysis, variants were reclassified according to the ACMG guidelines to incorporate the functional results (Supplementary Table S2) whereby de novo variants (PS1) that show deleterious effects from a well-established study (PS3) are classified as pathogenic and variants that are not confirmed de novo but show deleterious effects are classified as likely pathogenic. All truncating variants were classified as likely pathogenic as they are null variants in a gene where loss of function is a known mechanism of disease (PVS1). The group of variants that showed no change in the functional assay is highly heterogenous and will be addressed below.

### Segregation of *GABRB3* patients into cohorts

To determine if the diverse spectrum of clinical phenotypes was related to the functional change of GABA_A_ receptors, patients were assigned into cohorts based on the functional characterization of their variants. A total of 27 patients presented with variants observed to have GOF traits in the functional studies. A total of 47 patients presented with LOF variants; this included 11 patients with protein-truncating variants that would be expected to reduce total surface expression (haploinsufficiency). Finally, 11 patients were identified for which the functional analysis did not reveal significant changes, and these are investigated separately. The prevalence and severity of a range of different clinical indications were then compared.

### Key clinical features of patients with gain- vs. loss-of-function variants

Remarkable differences between the GOF and LOF patient cohorts were identified including age of seizure onset, types of seizures, severity of comorbidities, EEG features, behavioural abnormalities, and treatment responses (Supplementary Table S1).

#### Age of seizure onset

**Fig. 4:**
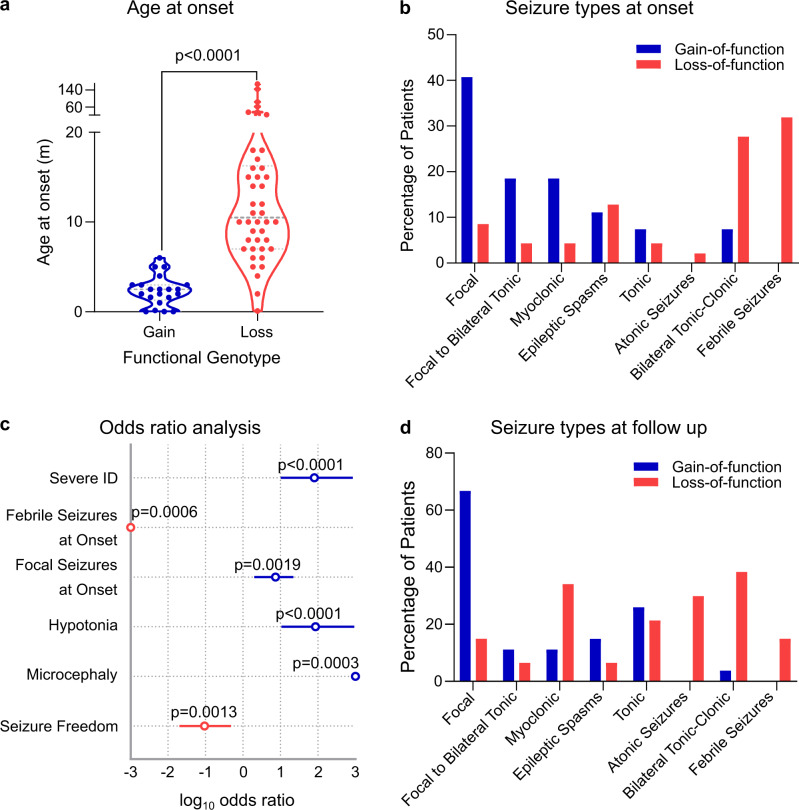
Clinical features of patients harbouring a LOF or GOF *GABRB3* variant. **a** Violin plot of age of seizure onset at gain-of-function (blue; *n* = 21 patients) and loss-of-function (red; *n* = 43 patients) variants. Dots represent individual data points, dotted lines represent median and quartiles. Two-sided unpaired Mann–Whitney *U* test, *p* = 1.1 × 10^−11^, *U* = 39). **b** Selected seizure types reported at onset for patients with gain-of-function (blue bars) and loss-of-function (red bars) variants. Percentage of patients reporting each seizure type is shown. **c** Odds ratio of key clinical outcomes in gain-of-function *vs*. loss-of-function variants with the centre circle showing the log_10_ odds ratio and 95 % confidence interval. Blue indicates significant enrichment in gain-of-function variants, and red indicates significant enrichment in the loss-of-function variants. For each indication, the total number of patients reporting were *n* = 18, 27, 27, 16, 11 and 19 gain-of-function patients, and *n* = 40, 47, 47, 27, 24 and 36 loss-of-function patients for severe intellectual disability, febrile seizures, focal seizures, hypotonia, microcephaly and seizure freedom, respectively. Fisher’s exact test, two-sided, *p* = 2.7 × 10^−8^, 2 × 10^−5^, 0.019, 3.5 × 10^−7^, 3 × 10^−4^ and 0.013, respectively. **d** Selected seizure types reported at follow-up for patients with a gain-of-function (blue bars) and loss-of-function (red bars) variants. Percentage of patients reporting each seizure type is shown. Source data are provided in the supplementary information (Supplementary Table S1).

Patients harbouring a GOF variant presented with their first seizure at a median age of 2.5 months ([CI:1.6-3], *n* = 21). This is significantly earlier compared to patients with LOF variants that had a median age 10.5 months ([CI:9-15], *n* = 43 Mann–Whitney *U* test *p* < 0.0001) (Fig. 4a).

#### Seizure types

For the 27 patients in the GOF cohort, the most frequently reported seizure type at onset were focal seizures (11/27, 41%), followed by focal to bilateral tonic-clonic and myoclonic seizures (both 5/27, 19%), (Fig. 4b, Supplementary Fig S8). At follow-up, seizure types included focal (18/27, 66%), tonic (7/27, 26%), and epileptic spasms (4/27, 15%). Surprisingly, febrile seizures were only identified in the LOF cohort (0/27 GOF cf. 15/47 LOF; Odds Ratio (OR) N.D, Fisher’s exact test *p* = 0.0006, Fig. 4c). For the 47 patients in the LOF cohort, the most frequently reported type of seizure at onset were febrile seizures (15/47, 32%), followed by bilateral tonic-clonic seizures (13/47, 28%) and epileptic spasms (6/47, 13%) (Fig. 4d). Seizure types at follow-up included bilateral tonic-clonic (18/47, 38%), myoclonic (17/47, 35%), atonic seizures (14/47, 29%), and tonic seizures (10/7 21%) (Fig. 4d, Supplementary Fig S9).

#### EEG

**Fig. 5:**
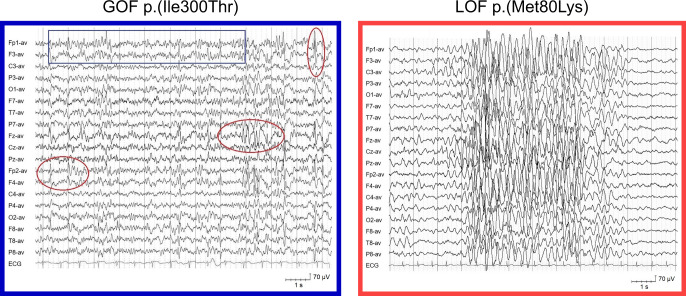
Representative electroencepholagram from patients harbouring a GOF or LOF variant. Interictal EEG showed a disorganized background activity with intermixed 12–15 Hz component (blue boxes) and multifocal epileptiform abnormalities (red circles) in patients with gain-of-function variants and normal/slightly delayed background activity, with generalized high amplitude 3–4 Hz activity/spike and slow waves (dotted lines) in patients with loss-of-function variants.

Patients harbouring a GOF variant presented with severely disorganized EEG background activity intermixed with rapid activity, and multifocal epileptiform abnormalities (Fig. 5). Patients in the LOF cohort had a normal or mild slowed EEG background, bursts of delta activity in the posterior regions or diffuse, and generalized spike and slow waves.

#### Intellectual disability

Severe intellectual disability (ID) was significantly more prevalent in the GOF cohort (17/18 cf. 7/40; OR 80.14 [CI 10-856], Fisher’s exact test *p* < 0.0001, Fig. 4c). In contrast, ID was reported to be mild (11/40), moderate (20/40) or severe (7/40) in the LOF cohort (Supplementary Fig. S10 and Table S3).

#### Behavioural abnormalities

There were no significant differences in the behavioural abnormalities reported in the GOF compared to the LOF cohort (6/9 cf. 27/36; OR = 0.19 [CI 0.15–2.9] Fisher’s exact test *p* = 0.6818). Abnormalities reported in the LOF cohort included autism spectrum disorder (6/36), autistic features (8/36) and ADHD (8/36) (Supplementary Fig. S11 and Tables S4, 5).

#### Other features

Hypotonia was more prevalent in patients with a GOF variant (15/16 cf. 4/27; OR 86 [CI 11-933] Fisher’s exact test *p* < 0.0001, Fig. 4c). Similarly, microcephaly was exclusively reported in patients with a GOF variant (6/11 cf. 0/24; OR N.D. Fisher’s exact test *p* = 0.0003, Fig. 4c, Supplementary Table S6).

#### Treatment response

Refractory epilepsy was significantly more prevalent in the GOF cohort, with only two patients becoming seizure free with treatment (2/19 cf. 20/36; OR 0.09 [CI 0.019–0.47], *p* = 0.0013, Fig. 4c). Patients in the LOF cohort had seizures that were responsive to treatment with valproate (8/19 seizure free in mono- or polytherapy), GABA enhancers (e.g., clobazam, phenobarbital and vigabatrin, 8/19) or levetiracetam (3/11). However, sodium channel blockers were poorly tolerated with only three patients (out of 15) experiencing a reduction in seizures and four experiencing worsened seizures (Table 1, Supplementary Tables S7–11). In contrast, GABAergic enhancers were not well tolerated for patients in the GOF cohort, with 2/13 experiencing a reduction in seizures and 3/13 with adverse effects (Table 1, Supplementary Tables S7–11).

**Table 1:** Drug responses in patients with loss- and gain-of-function *GABRB3* variants.

	Seizure free^a^	Seizure reduction	No effect	Worsening/adverse effects
*Loss-of-function GABRB3 patients*				
Sodium Channel Inhibitors^b^	12% (2/17)	5.9% (1/17)	59% (10/17)	24% (4/17)
GABAergic Enhancers^c^	42% (8/19)	5.3% (1/19)	47% (9/19)	5.3% (1/19)
Carbonic Anhydrase Inhibitors^d^	29% (2/7)	29% (2/7)	43% (3/7)	0% (0/7)
Valproate	44% (8/18)	22% (4/18)	28% (5/18)	5.6% (1/18)
Levetiracetam	30% (3/10)	10% (1/10)	60% (6/10)	0% (0/10)
Steroids/Ketogenic Diet	50% (2/4)	0% (0/4)	50% (2/4)	0% (0/4)
Overall	33% (25/75)	12% (9/75)	47% (35/75)	8% (6/75)
*Gain-of-function GABRB3 patients*				
Sodium Channel Inhibitors^b^	17% (1/6)	0% (0/6)	83% (5/6)	0% (0/6)
GABAergic Enhancers^c^	0% (0/12)	17% (2/12)	58% (7/12)	25% (3/12)
Carbonic Anhydrase Inhibitors^d^	20% (1/5)	20% (1/5)	40% (2/5)	20% (1/5)
Valproate	0% (0/6)	0% (0/6)	83% (5/6)	17% (1/6)
Levetiracetam	0% (0/9)	11% (1/9)	78% (7/9)	11% (1/9)
Steroids/Ketogenic Diet	20% (1/5)	0% (0/5)	80% (4/5)	0% (1/5)
Overall	7.0% (3/43)	9.3% (4/43)	70% (30/43)	14% (6/43)

^**a**^Includes when used either in monotherapy or polytherapy.

^b^Includes carbamazepine, oxcarbazepine, phenytoin, lacosamide and lamotrigine.

^c^Includes clobazam, clonazepam, nitrazepam, phenobarbital, stiripentol and vigabatrin.

^d^Includes sultiame and topiramate.

#### Inheritance

There was no significant difference in the mode of inheritance between the two cohorts. Patients were exclusively de novo in the GOF cohort and predominately de novo in the LOF cohort (23/25 cf. 34/44; OR ND, *p* = 0.011). Notably, eight of the ten inherited LOF variants were protein truncating variants.

#### Mortality

Two patients in the GOF cohort and one patient in the LOF cohort died from sudden unexpected death in epilepsy (SUDEP).

In summary, patients harbouring GOF variants have objectively more severe outcomes; they have a younger age of seizure onset, severe ID and are unlikely to respond to ASMs. In contrast, patients with LOF variants generally present with milder phenotypes such as febrile seizures followed by different types of generalized seizures, predominately myoclonic or atonic. Both GOF and LOF patients segregate into distinct clusters when analysed with either a two-step method or a multilayer perceptron (Supplementary Section S8). The validity of using the functional outcome as a predictor for clinical phenotype is elegantly illustrated by the two variants in the Thr281 residue mentioned above. The patient with the GOF p.(Thr281Ala) variant had seizure onset at less than a month of age, severe intellectual disability and is refractory to treatment. In contrast, the patient with the LOF p.(Thr281Ile) variant had seizure onset at seven months, moderate intellectual disability and responded to pentobarbital, stiripentol and valproate polytherapy (Supplementary Fig. S12). Hence, divergent functional outcomes offer an explanation to the conundrums of how a single, monogenic cause of disease can lead to a wide spectrum of clinical phenotypes, and why some patients are refractory to treatment.

### Variants that cause no change in GABA_A_ receptor function

Eleven patients contained 10 variants, five inherited and five de novo, that could not be classified as either GOF or LOF based on the functional studies. Generally, these patients had a mild to moderately severe phenotype that resembles the LOF patients (Supplementary Fig. S13 and section S10). This group could thus contain a mixture of variants that are benign, have incomplete penetrance or cause receptor dysfunction that is not affecting the chosen parameters in the functional assay. Reclassification according to the ACMG guidelines therefore leads to a variety of outcomes.

Two patients carried the inherited variants p.(Glu357Lys) and p.(Arg429Gln), that appear in the gnomAD database multiple times and these can therefore be classified as benign (BS2, BS3). A further three patients contained the inherited p.(Gly32Arg), p.(Val37Gly) and p.(Arg142Leu) variants. Neither the p.(Gly32Arg) or the p.(Val37Gly) variants are fully penetrant with unaffected carriers in the families, and can also be classified as benign (BS3, BS4). In relation to the Arg142 variant, another variant in this position, p.(Arg142His) is present in the gnomAD database, which is sufficient evidence to classify the p.(Arg142Leu) variant as likely benign. In support of the inherited variants being benign, all patients had seizures resolved by 12 years, 4 years and 18 months of age, respectively^4,13^. Hence, of the variants showing no functional effect, five inherited variants can all be classified as benign or likely benign and likely seen as population variants.

The remaining six patients contained five variants that were de novo, providing strong evidence for pathogenicity based on the ACMG guidelines (PS2). Of these, the functional data for the p.(Pro253Leu) variant stood out by being the only variant tested that showed a marked increase in desensitization kinetics (Supplementary Fig S14). While we elected not to formally categorize variants based on this receptor property, this would likely constitute a *de facto* LOF trait and the classification should remain likely pathogenic. Supporting this, the patient had an age of seizure onset of 11 months and bilateral tonic-clonic seizures, similar to other patients with LOF traits. For the remaining four variants (p.(Gln210His), p.(Leu310Ile), p.(Ser420Ile), and p.(Ser433Leu)), the combination of the de novo status with the functional analysis meant that these should be classified with a variant of unknown clinical significance (VUS) status.

### 2D structural mapping

**Fig. 6:**
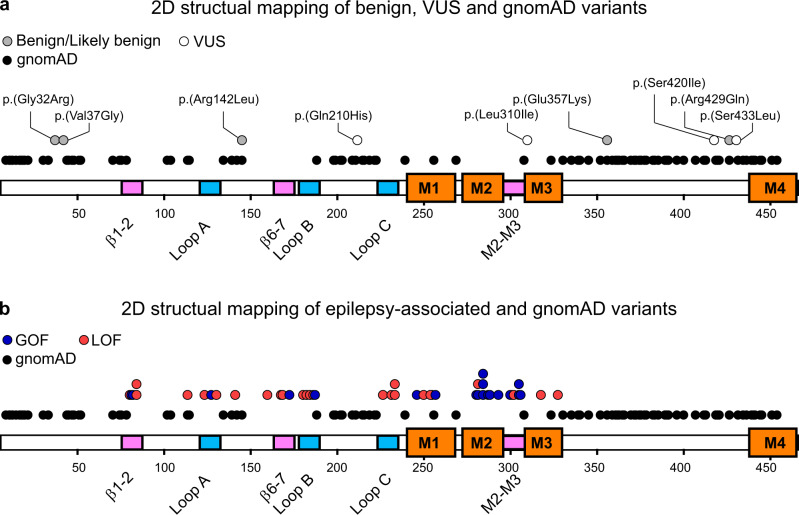
2D structural map of pathogenic and benign missense *GABRB3* variants. **a, b** Two-dimensional representation of a GABA_A_ receptor β3 subunit depicting binding loops (blue), coupling loops (pink) and four transmembrane regions (orange) is shown with a row of black dots indicating the location of benign variants in the gnomAD database. The upper row of dots indicates pathogenic or likely pathogenic GOF (blue), LOF (red) variants, benign or likely benign (grey) variants and VUS (white) variants. Source data are provided in the Source Data file.

Disease-causing and benign variants described in the gnomAD database were mapped onto the 2D structure of the β3 subunit^14^. Presumed benign variants at 92 residues were highly concentrated in locations of the protein sequence less critical for basic receptor function, including the signal peptide, initial α-helix, the β-sheets and disordered loops such as the large M3-M4 intracellular region (Fig. 6). Unsurprisingly, the inherited variants that caused no change in GABA_A_ receptor function and were classified as benign, are in regions rich in gnomAD database variants (Fig. 6a). This includes p.(Gly32Arg) and p.(Val37Gly) in the initial α-helix, p.(Arg142Leu) in a disordered extracellular loop, and p.(Glu357Lys) and p.(Arg429Gln) in the intracellular M3-M4 loop. The position of two VUS variants, p.(Ser420Ile) and p.(Ser433Leu), at amino acids that are not conserved across GABA_A_ receptor subunits, and in the intracellular M3-M4 loop regions suggest that these are also likely to be benign. The remaining two VUS variants, p.(Gln210His) and p.(Leu310Ile), are located in regions of high importance for receptor function and in amino acid positions of high degree of conservation across GABA_A_ receptor subunits. Hence, these two variants may affect receptor function in other parameters than those tested.

In sharp contrast, pathogenic GOF and LOF variants are overwhelmingly located in regions known to be critical for allosteric transduction of GABA-mediated receptor activation, including the GABA-binding site, the coupling loops that link the extracellular and transmembrane domains, and the pore-lining M2 transmembrane region along with the M1 and, to a lesser extent, the M3 region (Fig. 6b).

### 3D Structural Mapping

**Fig. 7:**
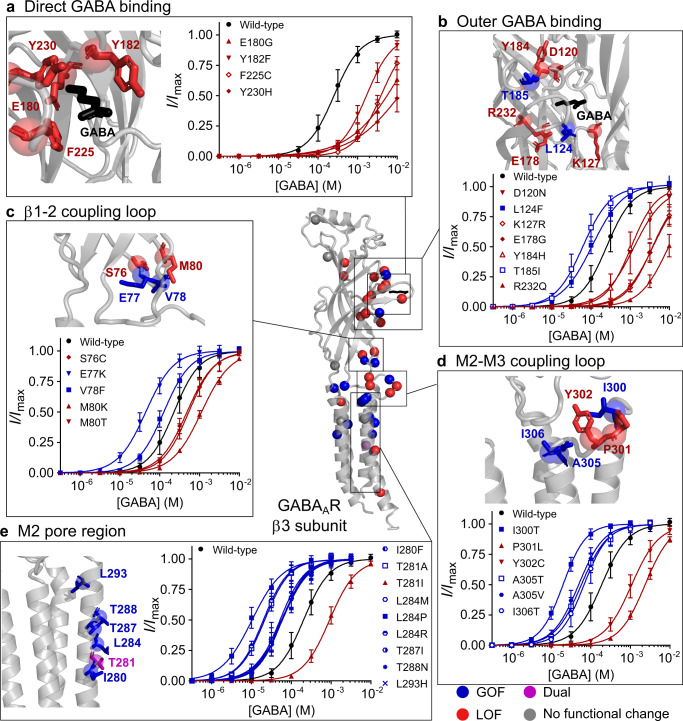
3D structural map and concentration–response curves. 3D structure of GABA_A_ receptor β3 subunit displaying the location of: gain-of-function (blue), loss-of-function (red) and dual (purple) variants, with the variants showing no functional change in grey. **a**–**e** Structure of the β3 subunit with the backbone coloured in grey and a close-up view of residues **a** directly binding GABA; **b** in the outer shell of GABA binding; **c** β1-2 coupling loop; **d** M2-M3 coupling loop and **e** M2 region with amino acid sidechains of residues containing variants in sticks. Red indicates loss-of-function, blue indicates gain-of-function variants and GABA is in black. Concentration–response curves for variants directly binding GABA. Dots represent mean ± s.d. and lines the fitted Hill equation. Wild-type concentration–response curves run on the same days as the variants are shown in black. Red indicates loss-of-function and blue indicates gain. For each panel, **a** *n* = 35, 12, 11, 12, 12 independent experiments at wild-type, E180G, Y182F, F225C, Y230H; **b** *n* = 34, 11, 12, 14, 13, 11, 13, 14 at wild-type, D120N, L124F, K127R, E178G, Y184H, T185I, R232Q; **c** *n* = 34, 12, 11, 12, 11, 10 at wild-type, S76C, E77K, V78F, M80K, M80T; **d** *n* = 36, 15, 11, 12, 10, 10, 24 at wild-type, I300T, P301L, Y302C, A305T, A305V, I306T; **e** *n* = 57, 12, 14, 11, 11, 12, 12, 11, 11, 11 at wild-type, I280F, T281A, T281I, L284M, L284P, L284R, T287I, T288N, L293H. Source data are provided in the Source Data file.

The GABA-binding site is located at the β3-α1 subunit interface of the extracellular domain (Fig. 1b). All residues within 4 Å of the GABA molecule that interact directly with GABA during the binding step, are LOF and markedly decrease GABA sensitivity (Fig. 7a). These residues are highly conserved and make tight interactions with GABA and alterations to these residues understandably result in LOF. In contrast, both GOF and LOF variants are found in residues that surround the GABA-binding site (Fig. 7b). The surrounding residues have important functions such as supporting the structural integrity of the binding pocket and initiating the first steps in the activation pathway. Hence, the observation that variants in these residues can lead to both GOF and LOF suggest one or the other of these functions are affected.

When the receptor transmits a signal of GABA binding to the ion channel pore and activates the channel, the coupling loops at the interface of the extracellular and transmembrane domains alter conformation. In particular, the extracellular domain β1-2 loop and transmembrane domain M2-M3 loop undergo large structural rearrangements during receptor activation^15^. Interestingly, variants at these coupling loops are a mixture of GOF and LOF, which is likely due to fact that they can either stabilize or destabilize the interactions and necessary conformational changes that occur between these loops (Fig. 7c, d).

Finally, the M2 transmembrane region lines the channel pore and is critical both in stabilizing receptor open and closed states, as well as interacting with ions passing through. Apart from the Thr281 residue, where both GOF and LOF variants were identified, variants within the M2 region were uniformly GOF (Fig. 7e). Many of these residues are aligned such that the sidechains are facing into the pore lumen, and several are congregated around the central Leu284 residue that is believed to hold the channel gate^15^. It is possible that the high number of GOF variants in M2 residues is due to these variants allowing greater flexibility of M2 helix to tilt into an open conformation, or act to destabilize the closed state. Nevertheless, this may not be a strict rule for all variants in this region, and it is conceivable that other variants could cause LOF by occluding the channel pore, stabilizing the central leucine in the closed state or repelling chloride ions.

Further detail on all structural motifs can be found in the supplementary information (Supplementary Figs. S4–7).

## Discussion

*GABRB3* variants that cause epilepsy have emerged at a steady rate in recent years, however, in most cases variants have been reported without any analysis of their functional effects. In the few cases where functional analysis was performed, it was invariably concluded that variants caused a LOF in the GABA_A_ receptor^2,4,16–18^. Similar conclusions were reported with variants identified in other *GABR* genes such as *GABRA1* and *GABRG2*^2,19–24^. Hence, an overall assumption has emerged dictating that epilepsy-associated *GABR* variants lead to LOF receptors, and that loss of GABAergic inhibition then leads to epilepsy. In support of this assumption, transgenic mouse models containing LOF variants display phenotypic characteristics that mimic the human observations^25,26^. However, recently, we reported two patients with *GABRB3* variants (p.(Glu77Lys) and p.(Thr287Ile)) showing hypersensitivity to vigabatrin and upon functional analysis identified that the variants resulted in GOF GABA_A_ receptors^27^. Furthermore, we identified six patients with GOF *GABRD* variants that shared common phenotypes of neurodevelopmental disorders with generalized epilepsy, behavioural issues, and various degrees of intellectual disability^28^.

These striking observations inspired us to perform functional analysis of all available *GABRB3* variants and correlate this with patient’s clinical genotype/phenotype presentations. Two distinct functional groups of pathogenic variants of approximately equal size were identified from the electrophysiological analysis: the novel GOF variants characterized by an increase in GABA sensitivity and the LOF variants characterized by a reduction in the GABA sensitivity. In fact, we observed that the key determining factor for the functional outcome of a variant was receptor sensitivity to GABA. Most de novo missense variants (92%) differed significantly from wild-type receptors for this parameter, and none caused LOF in maximal current amplitudes without also significantly affecting GABA sensitivity. One variant, p.(Pro253Leu), stood out from the rest by displaying a marked increase in desensitization kinetics (LOF trait) without affecting GABA sensitivity. As we elected not to formally investigate this parameter, this variant is not included in the genotype/phenotype correlations, but it is likely pathogenic and display LOF traits. For the nine variants for which no significant functional changes were observed, five are inherited and could be classified as benign or likely benign. Only four de novo variants were given a VUS classification yet based on structural extrapolations at least two of these are also likely to be benign. Hence, the vast majority of pathogenic *GABRB3* variants significantly affected the GABA sensitivity parameter.

By dividing patients into cohorts based on the functional outcome of their variant, clear distinct phenotypic differences emerged. Patients with GOF variants presented with their first seizure at a young age (typically 1–3 months) and a prominence of focal seizures and severe intellectual disability were key clinical indicators. Overall, these patients resemble the phenotype seen in other genetic DEEs, such as GOF *SCN2A* and *SCN8A*, where seizure onset is also within the first six months of life, cognitive outcome is poor and focal seizures are common^6,10^. Furthermore, GOF variants in *SCN1A* have recently been described in patients with early onset catastrophic DEEs^29^. There is also some phenotypic overlap with GOF *KCNH1* variants^30^. Patients with GOF variants almost universally presented with de novo variants, a feature that is likely associated with the severity of the clinical phenotype. The severity of DEE in patients with GOF variants also may explain why early familial studies of GABA_A_ receptor variants uncovered only LOF variants, while the more powerful next-generation sequencing has unearthed a sizeable cohort of de novo GOF variants.

In contrast, patients with LOF variants present with their first seizure at a later age (typically >1 year). Seizure type at onset were febrile seizures and bilateral tonic-clonic seizures. Other clinical indicators included mild-moderate intellectual delay. The phenotypic spectrum of these patients parallels those with LOF *SCN1A* variants. This includes disease initiation with febrile seizures that develops into generalized seizures and intellectual disability ranging from moderate to severe^31^. Phenotypic similarities between patients with LOF *SCN1A* and *GABRB3* variants likely reflect common neurological pathways^29,32^. While *GABRB3* LOF variants directly impair GABAergic neurotransmission, *SCN1A* LOF variants indirectly reduce GABAergic neurotransmission by inhibiting GABA release at GABAergic interneurons.

Finally, it is noteworthy that patients with *GABRB3* protein-truncating variants tend to have less severe outcomes than those with missense LOF variants, with absence seizures and frequent familial inheritance (Supplementary Fig S15, 16). Indeed, patients often inherit the truncating variant from an unaffected parent, suggesting that these variants are genetic risk factors rather than a Mendelian cause of disease^33^. A prematurely truncated β3 polypeptide thus appears to cause less havoc to GABAergic signalling than an impaired full-length subunit. This can be reconciled with the notion that a truncated β3 subunit lacks important transmembrane domains and is hence unable to assemble into functional receptors with other subunits. Patients with truncating variants therefore have an expected overall *GABRB3* expression level deficit (haploinsufficiency), however, all expressed receptors originating from the healthy allele are fully functional and maintain wild-type receptor GABA sensitivity. This contrasts to patients with missense variants, where up to approximately 75% of the receptors (50% heterozygote and 25% homozygote) would be expected to be malfunctional.

Response to ASMs differ substantially between the GOF and LOF cohorts. Patients that harbour GOF variants have poorer clinical outcomes as they are less likely to respond favourably to current treatments. Critically, these patients could have unfavourable outcomes with treatments that enhance GABAergic transmission^27^. Sodium channel inhibitors appear to have effect in some patients and could represent a foundation for further combination treatments that avoid GABAergic enhancers. Patients that harbour LOF variants have better clinical outcomes as they are more likely to respond favourably to treatment. ASMs that enhance GABAergic neurotransmission, such as clobazam and vigabatrin can be used to control seizures^34^. Indeed, approximately a third of patients with LOF variants exhibited seizure freedom with drugs such as clobazam, vigabatrin, levetiracetam and several other drugs either alone or in combination. The positive response to GABAergic drugs highlights the similarity of LOF *GABRB3* variants with LOF *SCN1A* variants and treatments that are contraindicated in patients with *SCN1A* variants, such as carbamazepine, could also exacerbate seizures in LOF *GABRB3* patients.

GOF and LOF epilepsy-causing variants have been shown to occur in many genes including those encoding sodium, calcium, and potassium channels and these have likewise been identified with distinct clinical phenotypes^32,35,36^. Although GOF variants in GABA_A_ receptors have been speculated, until now no one has convincingly demonstrated that this is a common phenomenon in DEE patients. Our findings thus add the major inhibitory receptor in the mammalian brain to a growing list of ion channels for which both GOF and LOF variants are observed in patients with DEE. As many classes of ion channels interact with each other in neuronal networks their pathogenetic pathways are likely to converge leading to resemblance between specific patient groups. Already, we observe that the clinical features of patients with GOF *SCN8A* variants resembles those with GOF *GABRB3* variants and LOF *SCN1A* resembles LOF *GABRB3* variants^31,32^.

**Fig. 8:**
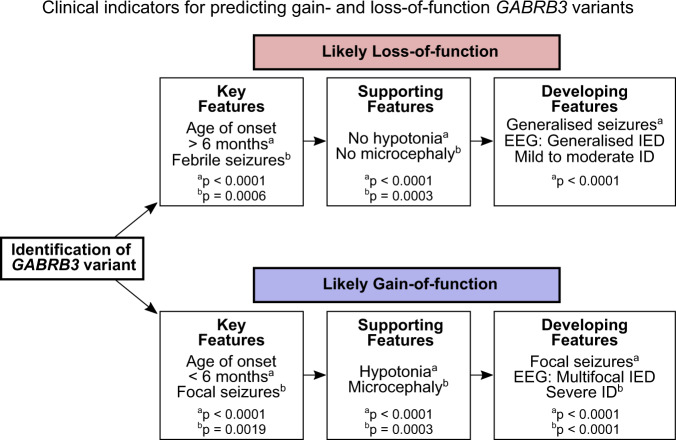
Flowchart for predicting LOF and GOF *GABRB3* variants based on clinical indicators. When a *GABRB3* missense variant is identified, there are several clinical features that can help predict whether the variant is LOF or GOF. Key features of LOF variants include an age of onset of 6 months or older and the presence of febrile seizures. Supporting features include a lack of hypotonia and microcephaly. As patients develop, LOF variants typically result in generalised seizures (includes atonic, myoclonic and bilateral tonic-to-clonic seizures), EEG with generalised interictal epileptiform discharges (IEDs) and ID in the normal or mild to moderate spectrum. For GOF variants, key features are focal seizures that present at an age of onset less than 6 months, and supporting features include hypotonia and microcephaly. As the patient ages, GOF variants feature focal seizures, EEG with multifocal interictal epileptiform discharges, and severe ID. If the patient presentation has a mixture of these indications, functional testing of the variant is needed to determine whether it has LOF or GOF traits. *p*-values comparing the clinical indicators for patients with LOF and GOF variants are included (also see Fig. 4).

To facilitate better outcomes for individual patients, the functional category should be established when patients with novel *GABRB3* variants are diagnosed. Unfortunately, it may not be possible to obtain functional analysis in many cases. Since patient phenotypes appear tightly linked to the functional outcome of the variants, clinical indicators can instead be used to predict the functional category with reasonable reliability. Initially, key features of the age of onset and the presence of focal or febrile seizures differentiate patients with a LOF or GOF variant, and the presence of hypotonia and/or microcephaly lend support to a GOF variant. As the patient ages, generalized seizure types, EEG signatures and severity of intellectual disability become additional predictors for whether the variant cause GOF or LOF. This information can be summarized into a flowchart that can aid clinicians predict whether a *GABRB3* variant has a GOF or LOF traits (Fig. 8). As most of the developing features won’t be available or properly quantifiable in infancy, the prediction power of the flowchart is best for patients aged 2 years or older. Ideally, this should also accommodate treatment options, however the scattered approach to treatment of *GABRB3* variants means that any analysis of treatment indications is substantially underpowered. There are simply too few patients to have been prescribed the same drug, let alone allowing for other considerations as the severity of the epileptic disorder or the time the drug is prescribed. Thus, there is an urgent need to properly diagnose patients with GABA_A_ receptor variants and thoroughly evaluate the responses to available treatments.

The neurological pathways that underlie the epileptic phenotype in GOF *GABRB3* variants currently remain obscure. Nevertheless, variants in genes within the GABAergic pathways support the notion of GOF variants in neurodevelopmental disorders. For instance, it has been shown that epilepsy can be caused by LOF *SLC6A1* GABA transporter variants that prolong GABAergic synaptic responses and increase tonic currents^37,38^. Loss of function *SLC12A5* variants that encode KCC2 chloride transporters that are critical for maintaining the chloride gradient and ensuring that GABAergic transmission is hyperpolarizing, also cause severe epileptic syndromes^39,40^. Furthermore, GABA_A_ receptor activity is integral in developmental processes including proliferation, migration, synaptic growth and integration and neuronal differentiation, where the receptor mediates neuronal depolarization^41^. Future studies are thus needed to unravel the precise details of the mechanism whereby the GOF variants cause DEE.

A range of different expression systems have been traditionally used to define the functional effect of GABA_A_ receptor variants including mammalian cells such as HEK293 and *Xenopus laevis* oocytes as described in the Methods. While the oocyte expression system is one of the gold-standard expression systems^24,33,42,43^, an oocyte is different from a mammalian cell. Yet, broadly similar values to ours, for GABA sensitivity and maximum current amplitudes have been reported at several residues including p.(Asp120Asn), p.(Leu170Arg), p.(Glu180Gly), p.(Ala305Val) and p.(Tyr302Cys) where expression of free subunits in mammalian cells were used^17,18^. These reports often indicate more severe effects on the GABA sensitivity than ours, most likely due to receptors with two variants affecting receptor function more than those with one^44^, nevertheless the overall change is similar. In contrast, for the p.(Thr288Asn) variant we observed GOF while a previous report concluded LOF traits^18^. However, it should be noted that the diazepam and Zn^2+^ responses utilized to differentiate ternary αβγ from binary αβ receptors differed markedly from the wild-type receptor in that study, suggesting a heterogenous receptor population. The concatenated approach circumvents the problem of heterogenous subtype expression of αβγ and αβ receptors with different GABA sensitivities that commonly occur in overexpression systems. Therefore, the concatenated expression system in oocytes is ideal for the determination of core parameters such as the GABA sensitivity, while mammalian cells are better for analysis of detailed receptors kinetics (see Methods).

While the concatenated approach has significant advantages, it could be envisioned that constraining receptor oligomerization may mask certain effects of individual variants. That said, there is little concrete evidence that single amino acid *GABRB3* variants contribute greatly to impaired receptor assembly or trafficking. For instance, an analysis of 32 benign and pathogenic *GABR* variants found that normal surface expression was maintained in all pathogenic variants^45^. Furthermore, the M3-M4 region thought to be critical for trafficking to the cell membrane contains a substantial number of benign variants in the gnomAD database. This includes the p.(Glu357Lys) variant that was previously reported as leading to trafficking defects^16^, but found as a benign variant in our analysis and has now been reported eight times in the gnomAD database. Likewise, for the inherited p.(Gly32Arg) and p.(Val37Gly) variants, previous reports suggested trafficking defects while we observe no functional changes, which is supported by the fact that these variants are not fully penetrant with unaffected carriers in the families. Thus, while the concatenated approach in an oocyte expression system may not detect subtle changes in surface expression, it appears less prone to detection of false positives.

In several instances, we conclude that a variant has GOF traits or is benign while previous analysis suggested LOF traits. In the case of the p.(Leu170Arg) and p.(Ala305Val) variants, while an increased GABA sensitivity was reported, reduced current amplitudes levels were interpreted as the primary parameter to conclude the variants were LOF^45^. However, this parameter can display a high degree of variation and should be analysed with caution as described in the Methods. When high numbers of variants, larger numbers of biological replicates and nonparametric statistical analysis with appropriately strict *p*-values are used, we observed no significant change to current amplitude levels, whereas GABA sensitivity strongly correlates with the patient phenotype. This underscores that current amplitude levels should only be used as an indicator for haploinsufficiency when changes are substantial.

The current study has some general limitations. Factors such as gender could not be investigated due to the number of patients (85 patients harbouring 63 distinct variants). Some traits were also not reported in all cases, but it made little difference whether they were included or removed in the statistical analysis. The functional assay we utilized displayed a remarkable ability to distinguish pathogenic variants from wild-type receptors thereby enabling robust classification of variants according to the ACMG criteria. Hence, our approach that compares a high number of variants with strictly defined statistical parameters is thus well-suited to interrogate the correlation of the genotype with the phenotype. Nevertheless, a cell in an in vitro expression system never fully reflects a neuron in an intact working brain, hence it cannot be excluded that the functional assay precludes detection of other important factors, such as developmental regulation of neuron-specific changes in receptor expression^27,46^.

In summary, we demonstrate that *GABRB3* variants can lead to GOF as well as LOF receptors and that the clinical phenotypes of patients are strongly linked to the functional outcome. Given the high sequence similarity and functional overlap between the various GABA_A_ receptor subunits, it is imperative to determine if a similar scenario exists for most if not all *GABR* genes whereby GOF variants are likely a common phenomenon. Our data marks a conceptual change in the understanding of the GABAergic system in epilepsy, recognizing that more severe epilepsy syndromes are found in patients with increased GABAergic activity. As such, the classical view of GABA_A_ receptor activity in the excitation/inhibition imbalance paradigm is an ill-suited theoretical base to understand epileptogenesis. As previous research has primarily focussed on how genetic variants that reduce GABAergic activity leads to epileptic disorders, it is now of paramount importance that we start to investigate how increased GABAergic activity leads to severe genetic epilepsies. Such research could also guide future drug discovery programs and aid the development of much-needed new treatment options which could potentially include negative allosteric modulators at GABA_A_ receptors.

## Methods

### Ethics

The study complies with all relevant ethical regulations as approved by the ethical committee of Region Zealand, Denmark (number SJ-91). Previously unpublished individuals (or parents, in the case of minors) signed informed consent. Oocyte extraction from *Xenopus* laevis frogs was performed following a protocol approved by the Animal Ethics Committee of The University of Sydney (AEC No. 2016/970) in accordance with the National Health and Medical Research Council of Australia code for the care and use of animals

### Study Design and Patient Selection

Previously published data from 74 patients^4,14,16^ (Supplementary Section 1) were combined with the unpublished data of 11 patients with pathogenic or likely pathogenic *GABRB3* variants. The new patients were collected through collaborations with epilepsy and genetic centres in Europe and Canada or in some cases facilitated via GeneMatcher^47^. Variants were absent from the gnomAD database at the beginning of the study and classified as either likely pathogenic or pathogenic using the American College of Medical Genetics (ACMG) guidelines^12,48^, apart from two inherited variants (p.(Glu357Lys) and p.(Arg429Gln)) that appeared in the gnomAD database eight and four times, respectively. Clinical information was collected by face-to-face interviews with patients and their families and from clinical charts. Data were compiled in a structured phenotype table. Transcript NM_000814.4/5 was used.

### Functional studies

GABA_A_ receptors, such as α1β3γ2, have a pentameric stoichiometry of (α1)_2_(β3)_2_(γ2)_1_. As *GABRB3* variants only affect one chromosomal allele, the most important receptor to investigate is the one that is heterozygous for the variant. Pentameric concatenated constructs represent a highly efficient method to obtain robust expression of such receptors in *Xenopus laevis* oocytes. As such, we used concatenated pentameric γ2-β3-α1-β3-α1 receptor constructs using human subunits^44,49^. To allow systematic alterations in only one of the two β3 subunits, a tetrameric construct, γ2-X-α1-β3-α1 where X denotes a “missing” β3 subunit position, was made. Wild-type and variant β3 subunits designed for cloning into the X position were purchased from GenScript (Singapore). A total of 54 β3 subunit variants were made, verified by sequencing and subcloned into the tetrameric concatenated construct using standard restriction digestion and ligation. cRNA of each concatenated receptor was produced from linearized cDNA using the mMessage mMachine T7 Transcription kit (Thermo Fisher).

Oocytes were obtained from *Xenopus laevis* frogs^49^. Briefly, ovarian lobes were removed from anesthetised adult female *Xenopus laevis* frogs. The lobes were then sliced into small pieces using a surgical knife and defolliculated by collagenase A treatment.

The cRNAs encoding 54 variant and one wild-type concatenated α1β3γ2 receptors were then injected into oocytes (25 ng/oocyte) and electrophysiological recordings were carried out using a custom-built two-electrode voltage-clamp apparatus^49^. All experiments were conducted in at least two batches of oocytes. To control for inter-day variation between oocyte batches, wild-type experiments were run along with variants on each experimental day. GABA concentration–response relationships (*n* = 10–17) were obtained by applying increasing concentrations of GABA and maximum current amplitudes (*n* = 22–39) were determined by applications of 10 mM GABA unless otherwise noted.

To determine the EC_50_ values of GABA concentration–response relationships, the Hill equation was fitted to peak GABA-evoked current amplitudes for individual oocytes:

1 $$I={{{{{{{\rm{Abs}}}}}}}.{I}}_{{\max }}\left(\frac{{[{{{{{\rm{A}}}}}}]}^{{{{n}}}_{{{{{{\rm{H}}}}}}}}}{{[{{{{{\rm{A}}}}}}]}^{{{{n}}}_{{{{{{\rm{H}}}}}}}}+{{{{{{{\rm{EC}}}}}}}_{50}}^{{{{n}}}_{{{{{{\rm{H}}}}}}}}}\right)$$

where Abs.*I*_max_ is the absolute maximum current, EC_50_ is the concentration eliciting half-maximum response, [A] is the ligand concentration and *n*_H_ is the Hill slope. Responses were then normalized to the fitted maximum response of individual curves. Individual oocytes for which a complete concentration–response curve was taken are recorded as a single determination (n) and the corresponding logEC_50_ value was calculated from the EC_50_ value. Final EC_50_ values were derived from fitting the Hill equation to all data for each construct (note that these values are used for information only not statistical analysis).

The average logEC_50_ for the wild-type construct (logEC_50_(wt)) for each experimental day was calculated and the ΔlogEC_50_ for each variant experimental determination on the same day was derived by the equation:

2 $$\Delta \,\log {{{{{\rm{E}}}}}}{{{{{{\rm{C}}}}}}}_{50}=\,\log {{{{{\rm{E}}}}}}{{{{{{\rm{C}}}}}}}_{50}(wt){-}\,\log {{{{{\rm{E}}}}}}{{{{{{\rm{C}}}}}}}_{50}$$

For statistical comparisons, the final ΔlogEC_50_ values for variants were determined from the mean (and error) of individual experiments.

Similarly, the maximum current (*I*_max_) was determined by the peak current elicited by 10 mM GABA at wild-type controls (Abs.*I*_max(wt)_) and variants (Abs.*I*_max_). The *I*_max_ for each single experiment at a variant was determined by the equation:

3 $${I}_{{{{{{\rm{max }}}}}}}=\,\frac{{{{{{{\rm{Abs}}}}}}}.{I}_{{{{{{\rm{max }}}}}}}}{{{{{{{{\rm{Abs}}}}}}}.I}_{{{{{{\rm{max }}}}}}({{{{{\rm{wt}}}}}})}}$$

Additional detail can be found in the supplementary Information (Section 2).

### Statistics

Curve fitting of GABA concentration–response relationships were performed by non-linear regression using GraphPad Prism 8.2.1. GABA sensitivities (ΔlogEC_50_ values) were compared with a one-way ANOVA and multiple corrections with Dunnett’s post-hoc test, whereas maximum current amplitudes (*I*_max_ values) were compared with one-way ANOVA (Kruskal–Wallis) and multiple corrections with Dunn’s post-hoc test. To compare age-of-seizure onset, a Mann–Whitney U test was performed comparing gain- and loss-of-function categories. For qualitative clinical outcomes including seizure types, degree of intellectual disability and seizure freedom, the odds ratio was compared with Fisher’s exact test. Additional detail can be found in the supplementary Information (Section 3).

For the cluster analysis, we ran both a two-step model and a neural network (multilayer perceptron) in SPSS 28.0 statistics program. A two-step model and multilayer perceptron analysed age of onset, severity of intellectual disability and all seizure types. To test the validity of our flowchart we again ran both a two-step model and a neural network (multilayer perceptron) in SPSS statistics program. We analysed the category of age of onset (i.e., greater or less than 6 months), hypotonia, focal and febrile seizures. In both cases of the multilayer perceptron, 70% of the data were used as a training indicator and 30% as testing.

### Experimental considerations for functional analysis

A key factor when developing a functional assay to assess receptor variants is to prevent the conclusion of false negatives and positives. Hence, there are several important points to consider when designing the functional assay. First and foremost, the functional parameters investigated should have an obvious translatability, meaning that significant changes for a variant in the in vitro setting is likely to translate into significant alterations in neuronal network activity in a living brain scenario. Secondly, conclusions based on the functional data should be performed within the framework of the ACMG criteria and current recommendations for assay validation^12,50^. Finally, the functional assay must be designed such that data are highly accurate and reproducible to account for the fact that even relatively minor alterations in the function of a GABA_A_ receptor can have substantial impact on neuronal activity.

In this study, we elected to focus on two functional parameters, GABA sensitivity and maximally evoked current amplitude of expressed receptors. The sensitivity to GABA represents a parameter with obvious translatability as this is an intrinsic property of a GABA_A_ receptor that determines the GABA-concentration range within which receptors are active. Maximally evoked current amplitudes, however, represents a parameter with less clear translatability. While a loss of GABA-evoked current amplitude could represent haploinsufficiency, an important consideration is that neuronal plasticity might lead to compensation with smaller effects in the living brain than may be predicted from the functional assay. Furthermore, a loss of current in an in vitro assay could be due both to changes in intrinsic receptor properties, such as gating efficiency (unaltered or decreased surface expression) and trafficking efficiency (decreased surface expression), but also various experimental factors, such as quality and concentration of the genetic material and the transfection or injection efficiency. Hence, firm conclusions based on loss of maximal current amplitudes should only be drawn when the changes are substantial.

To determine whether a variant is benign or pathogenic according to the ACMG criteria, we analysed the chosen functional parameters for a large number of variants including several that are found in the gnomAD database and presumed to be benign. This enabled us to validate the assay and confidently correlate functional changes to the clinical phenotype and utilize the results to classify variants according to ACMG criteria^12,50^.

Finally, we took several steps to ensure accurate and reproducible outcomes were obtained in the functional assay. These included:

1. Using a concatenated receptor approach expressed in the *Xenopus* oocyte expression system. There are two gold-standard in vitro methodologies in common use for assessing GABA_A_ receptor variants: patch clamp electrophysiology using mammalian, most commonly HEK293 cells, and two-electrode voltage-clamp electrophysiology using *Xenopus laevis* oocytes, each with its own set of advantages and disadvantages^51^. While the patch clamp system allows for more detailed analysis of properties such as receptor activation and desensitization kinetics, the oocyte system is highly robust and allows for greater flexibility with respect to genetic material. For our studies, the flexibility in design of genetic material was critical in investigating uniform populations of receptors heterozygous for the variant using concatenated constructs. The α1β3γ2 GABA_A_ receptor subtype is the major subtype expressed in the mammalian brain where it mediates the well-known phasic inhibition attributed to synaptic GABA_A_ receptors. Commonly, researchers have used free GABA_A_ receptor subunits to assess function, which involves injecting or transfecting mixtures of three different subunit types. However, this approach will lead to the expression of heterogeneous populations of receptors, e.g. binary α1β3 receptors along with ternary α1β3γ2 receptors, in all expression systems. As these receptor combinations have different functional characteristics this causes a range of problems for measuring robust receptor activity, but more critically, contributes to GABA sensitivity measurements, artificially increasing variance and potentially lead to erroneous conclusions. By using concatenated receptors, this issue is avoided as the technique allows for expression of a uniform population of receptors that only contains one receptor arrangement^44^. Furthermore, it is possible to design constructs with only one *GABRB3* variant, which then enables measurements of receptors that are heterozygous for the variant.

2. Parallel electrophysiological recordings on a high throughput recording apparatus. When comparing results from different batches of cells whether it be oocytes or mammalian cells, or when recording from different days, the batch-to-batch or inter-day variation can lead to false negatives and positives. Variability between days and batches can produce significant differences that are larger than when comparing variants and wild-type controls. We addressed this by performing experiments of variant receptors with wild-type receptors in parallel with equal number of replicates on the same day and normalized the GABA sensitivity and maximum currents of variants to wild-type receptors for each recording day. This circumvented the influence of batch-to-batch and inter-day variations.

3. Using high number of replicates to attain sufficient statistical power. It is well known that relatively small changes in GABA sensitivity at GABA_A_ receptors lead to profound physiological effects. For instance, clinically relevant doses of benzodiazepines elicit a modest ~2-fold increase in GABA sensitivity yet this leads to profound neural effects, including hypnosis, sedation, muscle relaxation and anxiolysis. This demonstrates that the brain is acutely sensitive to even very small changes in GABA sensitivity, but also presents a technical challenge. To robustly detect relatively small changes in GABA sensitivity, we measured a minimum of ten replicates in a minimum of two batches of oocytes. For maximum current measurements, an even higher number of replicates was necessary as injections of cRNA into oocytes (or transfection of cDNA into mammalian cells for that matter) inherently lead to very large cell-to-cell and day-to-day variations. We therefore performed a minimum of 20 replicates for all experiments.

4. Using appropriate statistical tests and stringent *p*-values. The logEC_50_ values follow normal distributions allowing for the use of an ANOVA with Dunnett’s corrected post-hoc test. Receptor expression levels, however, does not follow a normal distribution and were therefore compared with a ranked statistical test. In both cases, a stringent *p*-value of *p* < 0.0001 was used to avoid false positives.

### Reporting summary

Further information on research design is available in the Nature Research Reporting Summary linked to this article.

## Supplementary information

Supplementary Information

Supplementary Table 1

Reporting Summary

## Data Availability

De-identifiable clinical data for 85 patients relevant for all statistics and conclusions in this manuscript has been provided in the supplementary information (Supplementary Table S1). Data for 74 of these patients were previously published^4,14,16^. Potential additional clinical information in our *GABRB3* database for the remaining 11 patients that does not breach privacy will be made available to those eligible upon request to the corresponding authors. Eligibility requires an approved study protocol. For functional data, the peak current amplitudes that were used to analyse logEC_50_ values and *I*_max_ current amplitudes are provided in the Source Data file. This also contains the original source filenames should requests be made for source trace files. Raw source trace files will be stored for a minimum of seven years at the University of Sydney and are available upon request to the corresponding authors. Source data are provided with this paper.

## Source data

Source Data file
