# Supplementary material for: Gain-of-function and loss-of-function GABRB3 variants lead to distinct clinical phenotypes in patients with developmental and epileptic encephalopathies
Source: Nat Commun. 2022 Apr 5;13:1822. doi: 10.1038/s41467-022-29280-x (PMC8983652; doi:10.1038/s41467-022-29280-x)
Supplement: Supplementary file 1 — Supplementary Information [file 41467_2022_29280_MOESM1_ESM.pdf]

Supplementary Materials for

Gain-of-function and loss-of-function *GABRB3* variants lead to distinct clinical phenotypes in patients with developmental and epileptic encephalopathies.

Nathan L Absalom†, Vivian W Y Liao†, Katrine M H Johannesen, Elena Gardella, Julia Jacobs-Le Van, Gaetan Lesca, Zeynep Gokce-Samar, Alexis Arzimanoglou, Shimriet Zeidler, Pasquale Striano, Pierre Meyer, Ira Benkel-Herrenbrueck, Inger-Lise Mero, Jutta Rummel, Mary Chebib, Rikke S Møller* and Philip K Ahring*.

†These authors contributed equally to this work, *Corresponding authors.

*Corresponding author. Email: philip.ahring@sydney.edu.au, rimo@filadelfia.dk

Contents

Section 1: Search strategy for epilepsy-associated *GABRB3* variants ... 3
Section 2: Detailed methods for functional evaluation of concatenated receptor constructs ... 4
Supplementary Figure S1 ... 7
Section 3: Detailed methods for evaluation of clinical phenotypes ... 8
Section 4: Detailed results of individual patient phenotypes ... 9
Supplementary Table S1. ... 9
Section 5: Detailed results of electrophysiological functional assays ... 10
Supplementary Table S2. ... 10
Supplementary Figure S2 ... 12
Supplementary Figure S3 ... 13
Supplementary Figure S4 ... 15
Supplementary Figure S5 ... 17
Supplementary Figure S6 ... 19
Supplementary Figure S7 ... 21
Section 6: Detailed seizure types of gain- and loss-of-function variants ... 22
Supplementary Figure S8 ... 22
Supplementary Figure S9 ... 23
Section 7: Detailed analysis of intellectual disability at gain- and loss-of-function variants ... 24
Supplementary Figure S10 ... 24
Section 8: Detailed comorbidities at gain- and loss-of-function variants ... 25
Supplementary Figure S11 ... 25
Supplementary Table S4. ... 26
Supplementary Table S5. ... 27
Supplementary Table S6. ... 28
Section 9: Drug reactions at gain- and loss-of-function variants ... 29
Supplementary Table S7. ... 29
Supplementary Table S8. ... 30
Supplementary Table S9. ... 31
Supplementary Table S10. ... 32
Supplementary Table S11. ... 33
Section 10. Opposite molecular and clinical phenotypes of variants at the T281 residue. ... 34
Supplementary Figure S12 ... 34
Section 11: Extended results and discussion of variants with no functional change ... 36
Supplementary Figure S13 ... 37
Section 12. Increased deactivation kinetics of the p.(Pro253Leu) variant ... 38
Supplementary Figure S14 ... 38
Section 13. Characteristics of patients with protein truncating variants. ... 39
Supplementary Figure S15 ... 39
Supplementary Figure S16 ... 40

Section 1: Search strategy for epilepsy-associated *GABRB3* variants

Our search strategy to determine the current consensus of the prevalence and functional phenotype of $GABA_A$ receptor variants began with a literature search in PubMed. We searched the terms "GABA" "variants" and "epilepsy" and restricted the search to peer-reviewed articles. Of the 188 identified articles, missense $GABA_A$ receptor variants were consistently described in different types of epilepsy and refractory epilepsy. Functional analysis almost universally described the phenotype as loss-of-function, including the two most recent reviews of the literature (*1, 2*). Although contrasting experimental results were published in two separate articles describing variants that increased the sensitivity of $GABA_A$ receptors associated with epilepsy, both articles concluded that increased desensitization properties and thus loss-of-function molecular phenotypes were underlying the disorder (*3, 4*). A single article from our group claimed that variants responsible for adverse drug reactions to vigabatrin were associated with gain-of-function *GABRB3*, however this study did not investigate any genotype/phenotype correlations (*5*).

Of the 19 subunits that make up the $GABA_A$ receptor family, variants have been identified in 11 subunits with varying frequency. Of these, variants are found in the *GABRB3* subunit appears with a particularly high prevalence. Hence, the *GABRB3* subunit is a highly suitable target for conducting an exhaustive genotype/phenotype study. Therefore, we searched Pubmed with the search term "*GABRB3*" for articles with the latest at 1st February 2021. Of the 380 articles, 26 articles described the identity and clinical details of 36 variants (*5-30*), and of these some type of functional analysis was performed for 12 variants (*8, 16, 19, 20, 26, 27, 30*). We subsequently identified a further 49 patients, the majority described in detail elsewhere (*31*).

The limited functional testing performed for *GABRB3* variants is problematical for several reasons. First, the existence of a variant in a patient does not automatically confirm that this is the root of the disease, as a variant might not change $GABA_A$ receptor function. Second, with only a limited number of variants tested, there is a risk of bias in the conclusions regarding the functional effects of variants, e.g., one-size-fits-all-conclusions may re-enforce pre-existing paradigms. Finally, the low numbers of tested variants did not enable genotype/phenotype correlations of the diverse clinical presentations of patients with epilepsy-associated *GABRB3* variants. We therefore sought to determine the functional phenotype of all known *GABRB3* variants to determine genotype/phenotype correlations and treatment responses, with the aim of accurate diagnosis, understanding disease progression and guiding superior treatment strategies for these patients.

Section 2: Detailed methods for functional evaluation of concatenated receptor constructs

Concatenated receptor constructs were created in the order γ2-β3-α1-β3-α1 with linker sequences as follows: γ2-$(AGS)_5$-β3-$(AGS)_2$ASLGS$(AGS)_3$-α1-AGT$(AGS)_5$-β3-$(AGS)_4$ATG$(AGS)_4$-α1 (*32*). Variants were placed in the second position of the concatenated receptor (*i.e.,* the β3 subunit that lies between the γ2 and α1 subunits in the heteropentamer) while all other subunits remained as wild-type. When cRNAs of the receptor constructs are injected into *Xenopus laevis* oocytes, the receptor assembles in the order γ2-β3-α1-β3-α1 in an anti-clockwise orientation when viewed extracellularly (*32, 33*).

cDNA and cRNA

E. coli bacteria were used as hosts for plasmid amplification, and plasmid purifications were performed with Qiagen plasmid purification kit. Complementary RNA (cRNA) was produced from linearised cDNA using the mMessage mMachine T7 Transcription kit (Invitrogen) according to the manufacturer's instructions. Due to the large size of the pentameric constructs (> 10 kb), to ensure maximal complete RNA transcripts, the guanosine triphosphate concentration was increased to give a final cap analogue ($m^7G(5')ppp(5')G$, an mRNA cap to promote eukaryote RNA translation) to guanosine triphosphate ratio of 2:1.

Expression of $GABA_A$Rs in Xenopus laevis oocytes

Oocytes were obtained and prepared as previously described (*5*). Briefly, ovarian lobes were removed from anesthetised adult female *Xenopus laevis* frogs following a protocol approved by the Animal Ethics Committee of The University of Sydney (AEC No. 2016/970) in accordance with the National Health and Medical Research Council of Australia code for the care and use of animals. The lobes were then sliced into small pieces using a surgical knife and defolliculated by collagenase A treatment. Stage V and VI oocytes were injected with 25 ng of cRNA encoding the $GABA_A$R pentamers and incubated for 2-4 days at 18°C in modified ND96 solution (96 mM NaCl, 2.0 mM KCl, 1 mM $MgCl_2$, 1.8 mM $CaCl_2$, 5 mM HEPES, 2.5 mM sodium pyruvate, 0.5 mM theophylline, and 100 µg/mL gentamicin; pH 7.4). Then the oocytes were subjected to two-electrode voltage clamp electrophysiology. To enable direct comparisons, data for the wild-type and variant β3 receptors were obtained in a parallel manner on each experimental day.

Electrophysiological recordings using two-electrode voltage clamp

Oocytes were placed in a recording chamber and continuously perfused with ND96 solution (96 mM NaCl, 2 mM KCl, 1 mM $MgCl_2$, 1.8 mM $CaCl_2$, and 10 mM HEPES; pH 7.4). Pipettes were backfilled with 3 M KCl with open pipette resistances ranged from 0.4 to 2 MΩ when submerged in ND96 solution. Cells were voltage-clamped at a holding potential of -60 mV using an Axon GeneClamp 500B amplifier (Molecular Devices, LLC, Sunnyvale, CA, USA). Amplified currents were filtered at 20 Hz by a four-pole low-pass Bessel filter (Axon GeneClamp 500B), digitized by a Digidata 1440A (Molecular Devices), and sampled at 200 Hz as well as analysed on a personal computer using the pClamp 10.2 suite (Molecular Devices). Responses to individual applications were collected as episodic traces following triggering events. GABA was dissolved in ultrapure water (3.16 M stock solution), 8-10 concentrations of GABA solutions were applied to oocytes in increasing concentrations with a

flow rate of 2.0 mL/min via a glass capillary. Each application lasted 25 or 35 s, and the application system ensured rapid solution exchange (in the order of a few seconds).

GABA concentration-response curve and maximum GABA-elicited current amplitude

To evaluate the function of each variant receptor, great care was taken to control for the batch-to-batch variability of concentration-response curve parameters from *Xenopus* oocytes. At all times, EC_{50} values and maximum peak current amplitudes were determined for the wild-type concentration-response curves on the same day as variant receptors.

Concentration-response curves were constructed by measuring the peak currents elicited by increasing half log concentrations 8-10 applications of GABA following 7 applications of control (3 applications of 50 μM before and after an application of 3.16 mM GABA). The maximum current for each individual oocyte was the peak current elicited at the highest concentration of GABA applied (10 mM) (Fig. S1A). To determine their EC_{50} values, the Hill equation was fitted to peak GABA-evoked current amplitudes for individual oocytes:

$$I = Abs.I_{max}(\frac{[\mathrm{A}]^{\mathrm{n_H}}}{[\mathrm{A}]^{\mathrm{n_H}} + \mathrm{EC_{50}}^{\mathrm{n_H}}})$$

Where $Abs.I_{\max}$ is the absolute maximum current, EC_{50} is the concentration eliciting half-maximum response, [A] is the ligand concentration and n_H is the Hill slope. Individual oocytes for which a complete concentration-response curve was taken are recorded as a single n. Responses were normalized to the fitted maximum response of individual curves. The EC_{50} is derived from fitting the Hill equation to all data. For statistical comparisons, the $\log EC_{50}$, $Abs.I_{\max}$ and n_H values were determined from the mean (and error) derived from fitting curves to individual experiments.

Final concentration-response curve datasets consisted of data from a minimum of n = 10 individual oocytes performed on a minimum of two independent batches of oocytes (*i.e.,* > two *Xenopus laevis* frogs) with equal or similar number of n for each oocyte batch. Likewise, final maximum response determinations were from a minimum of n = 22 individual oocytes.

The average $\log EC_{50}$ ($\log EC_{50(wt)}$) for the wild-type construct was determined and the $\Delta \log EC_{50}$ for each variant experiment was determined by the equation:

$$\Delta \log EC_{50} = \log EC_{50(wt)} - \log EC_{50}$$

Calculation is exemplified in Fig. S1B.

This enabled statistical comparison between variants measured on different batches of oocytes, whereby the $\Delta \log EC_{50}$ values were compared with an ANOVA and Dunnett's post-hoc test.

Similarly, the normalized maximum current (I_{max}) was determined by the peak current elicited by 10 mM GABA ($Abs.I_{\max}$) at wild-type controls ($Abs.I_{\max(wt)}$). The $I_{\max}$ for each single experiment at a variant was determined by the equation:

$$I_{\max} = \frac{Abs.I_{\max}}{Abs.I_{\max(\mathrm{wt})}}$$

This enabled statistical comparisons between variants, whereby the normalized I_{max} values were compared with a non-parametric ANOVA and Dunn's corrected post-hoc test.

Gain, or loss-of-function was determined by the $\Delta logEC_{50}$ values, where a significant shift in the concentration-response curve to the right defined a loss-of-function (Fig. S1C) and a significant shift to the left defined gain-of-function (Fig. S1D, Table S2). A comparison of I_{max} values was then performed, where a significant reduction was considered a loss-of-function.

To ensure robust associations and an adequately strong effect size, variants were categorized as loss- or gain-of-function variants when the p-value of the $\Delta logEC_{50}$ or normalized I_{max} values was <0.0001.

Supplementary Figure S1.

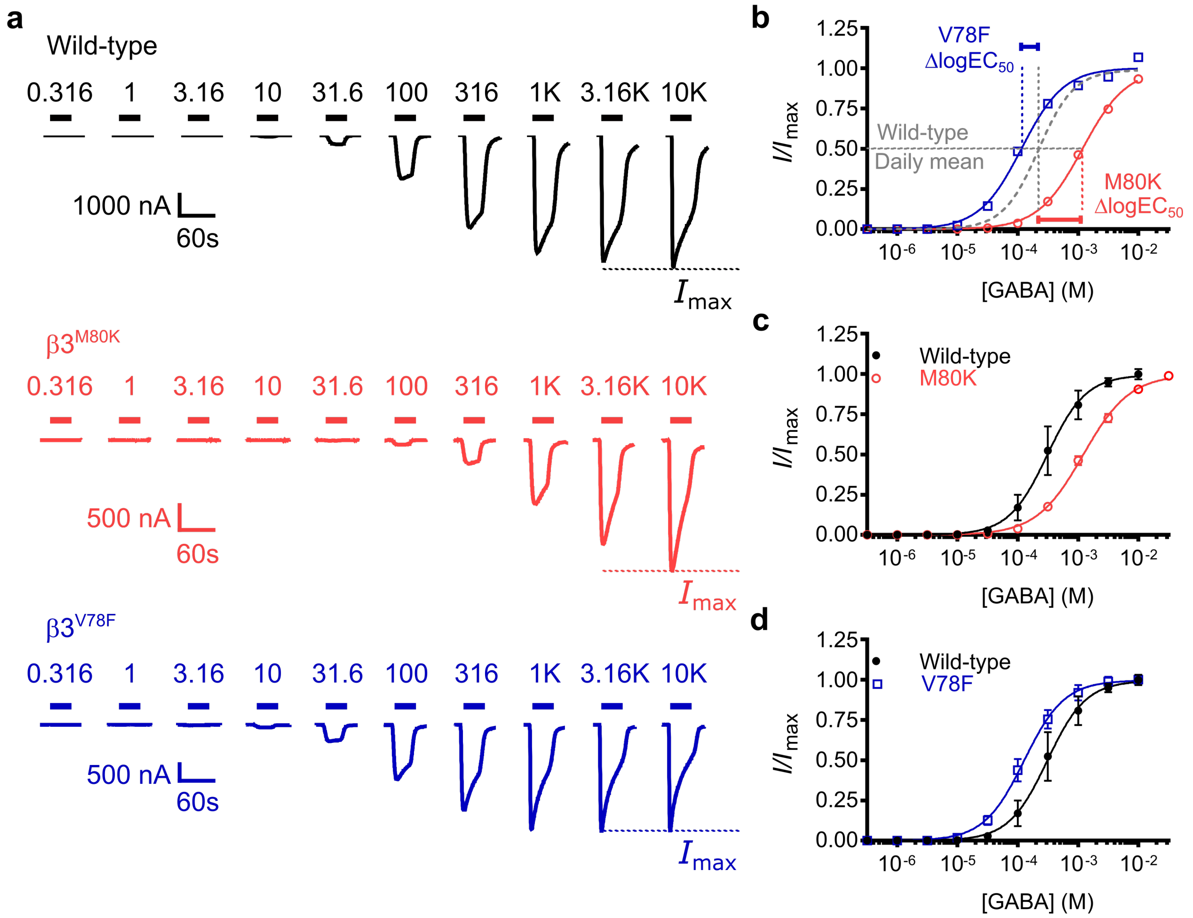

Section 3: Detailed methods for evaluation of clinical phenotypes

Statistical Comparisons

Where possible, clinical data were compared between loss- and gain-of-function variants with an odds ratio and Fischer's exact test. Age of onset was compared with a Mann-Whitney U-test. Variants with no functional change were excluded from statistical comparisons for two reasons: (1) Low numbers of patients which made associations unreliable and (2) the higher possibility that variants in other genes influenced the patient phenotype for this group.

A p-value of $p < 0.01$ was considered significant for these statistical analyses.

Handling of Incomplete Data

For statistical comparisons, incomplete data where values were unreported for individual patients was handled in the following manner:

Patients were excluded from the analysis of the age of seizure onset when the data were not recorded.

Patients were considered positive for seizure types when recorded, otherwise were considered negative.

For severe intellectual disability, hypotonia, microcephaly, seizure freedom, movement, vision impairment, speech issues, digestive issues, scoliosis and pyramidal and extrapyramidal signs, patients were excluded from the analysis when the data were not recorded.

Section 4: Detailed results of individual patient phenotypes

Information for individual patients was collated and organized into a table with gain, or loss-of function defined.

Supplementary Table S1.

Summary of clinical features are found in a separate Excel file.

Section 5: Detailed results of electrophysiological functional assays

Functional assays were performed to determine if variants significantly altered either the sensitivity to GABA or the maximum current amplitudes of oocytes expressing variant GABA$_A$ receptor constructs. A total of 54 missense variants were tested and a summary analysis table of the two parameters is shown below (Table S2).

Supplementary Table S2.

Fitted electrophysiological parameters for each variant.

Variant	Cat	EC$_{50}$ (μM)	ΔlogEC$_{50}$ ± s.d	N	p-value	Normalized I_{max} ± s.d	n	p-value	ACMG Classification
Wild-type	NA	221	-0.00 ± 0.10	135	NA	1.00 ± 0.32	256	NA	
Del ex1-3	Loss		N.D.			N.D.			Likely Pathogenic
p.(Trp2*)	Loss		N.D.			N.D.			Likely Pathogenic
p.(Gly3fs*26)	Loss		N.D.			N.D.			Likely Pathogenic
p.(Gly32Arg)	None	190	0.02 ± 0.11	10	0.9997	0.95 ± 0.29	27	0.9999	Uncertain
p.(Val37Gly)	None	140	0.16 ± 0.15	15	0.0004	0.93 ± 0.30	33	0.9999	Uncertain
p.(Ser76Cys)	Loss	480	-0.39 ± 0.10[*]	12	<0.0001	0.65 ± 0.27[*]	27	0.0001	Pathogenic
p.(Glu77Lys)	Gain	45	0.64 ± 0.11[*]	11	<0.0001	0.66 ± 0.27	24	0.0007	Pathogenic
p.(Val78Phe)	Gain	130	0.40 ± 0.21[*]	12	<0.0001	0.81 ± 0.50	24	0.1905	Pathogenic
p.(Met80Lys)	Loss	1100	-0.56 ± 0.16[*]	11	<0.0001	0.39 ± 0.19[*]	24	<0.0001	Pathogenic
p.(Met80Thr)	Loss	540	-0.45 ± 0.08[*]	10	<0.0001	0.69 ± 0.24	26	0.0041	Pathogenic
p.(Asn110Asp)	Loss	350	-0.23 ± 0.08[*]	15	<0.0001	0.99 ± 0.49	33	0.9999	Pathogenic
p.(Arg111*)	Loss		N.D.			N.D.			Likely Pathogenic
p.(Gln115*)	Loss		N.D.			N.D.			Likely Pathogenic
p.(Asp120Asn)	Loss	1200	-0.82 ± 0.22[*]	11	<0.0001	0.88 ± 0.55	23	0.9999	Pathogenic
p.(Leu124Phe)	Gain	110	0.50 ± 0.18[*]	12	<0.0001	0.85 ± 0.42	23	0.9999	Pathogenic
p.(Lys127Arg)	Loss	3 600	-1.11 ± 0.16[*]	14	<0.0001	0.28 ± 0.12[*]	24	<0.0001	Pathogenic
p.(ins138_139His)	Loss	340	-0.28 ± 0.14[*]	15	<0.0001	0.83 ± 0.34	30	0.9999	Pathogenic
p.(Arg142Leu)	None	160	0.10 ± 0.10	10	0.6582	0.86 ± 0.36	27	0.9999	Uncertain
p.(Thr157Met)	Loss	350	-0.25 ± 0.11[*]	11	<0.0001	1.09 ± 0.37	24	0.9999	Likely Pathogenic
p.(Leu165Gln)	Loss	2 800	-1.01 ± 0.10[*]	14	<0.0001	0.20 ± 0.08[*]	24	<0.0001	Pathogenic
p.(Arg166Ser)	Loss	400	-0.28 ± 0.15[*]	15	<0.0001	1.10 ± 0.34	33	0.9999	Pathogenic
p.(Leu170Arg)	Gain	110	0.26 ± 0.11[*]	12	<0.0001	0.90 ± 0.32	27	0.9999	Pathogenic
p.(Glu178Gly)	Loss	3 800	-1.06 ± 0.15[*]	13	<0.0001	0.74 ± 0.33	24	0.0476	Likely Pathogenic
p.(Glu180Gly)	Loss	3 200	-1.73 ± 0.12[*]	12	<0.0001	0.09 ± 0.05[*]	27	<0.0001	Pathogenic
p.(Tyr182Phe)	Loss	1 700	-0.91 ± 0.13[*]	11	<0.0001	0.85 ± 0.49	27	0.9999	Pathogenic
p.(Tyr184His)	Loss	1 100	-0.72 ± 0.11[*]	11	<0.0001	0.78 ± 0.32	26	0.3590	Pathogenic
p.(Thr185Ile)	Gain	62	0.59 ± 0.19[*]	13	<0.0001	0.73 ± 0.44	24	0.0048	Pathogenic
p.(Arg194*)	Loss		N.D.			N.D.			Likely Pathogenic
p.(Gln210His)	None	140	0.13 ± 0.17	14	0.039	0.98 ± 0.32	30	0.9999	Uncertain
p.(Phe225Cys)	Loss	3 900	-1.27 ± 0.09[*]	12	<0.0001	0.53 ± 0.26[*]	24	<0.0001	Pathogenic
p.(Tyr230His)	Loss	12 000	-1.67 ± 0.17[*]	12	<0.0001	0.04 ± 0.03[*]	24	<0.0001	Pathogenic
p.(Arg232Gln)	Loss	8 800	-1.50 ± 0.10[*]	14	<0.0001	0.50 ± 0.24[*]	24	<0.0001	Pathogenic
p.(Arg232Pro)	Loss		N.D.			0.01 ± 0.01[*]	22	<0.0001	Pathogenic
p.(Arg232*)	Loss		N.D.			N.D.			Likely Pathogenic
p.(Tyr245His)	Gain	77	0.37 ± 0.14[*]	12	<0.0001	0.81 ± 0.31	24	0.9999	Likely Pathogenic
p.(Gln249Lys)	Loss	690	-0.53 ± 0.14[*]	11	<0.0001	0.88± 0.31	26	0.9999	Pathogenic
p.(Pro253Leu)	None	290	-0.02 ± 0.11	12	0.9996	0.81 ± 0.50	24	0.1535	Uncertain[e]
p.(Ser254Phe)	Gain	110	0.24 ± 0.09[*]	11	<0.0001	1.10 ± 0.40	24	0.9999	Pathogenic
p.(Leu256Gln)	Gain	81	0.47 ± 0.17[*]	11	<0.0001	0.96 ± 0.41	24	0.9999	Pathogenic
p.(Ile280Phe)	Gain	58	0.65 ± 0.13[*]	12	<0.0001	1.01 ± 0.27	24	0.9999	Pathogenic
p.(Thr281Ala)	Gain	21	0.90 ± 0.07[*]	14	<0.0001	1.07 ± 0.38	24	0.9999	Pathogenic
p.(Thr281Ile)	Loss	910	-0.55 ± 0.13[*]	11	<0.0001	0.63 ± 0.27	24	0.0003	Pathogenic
p.(Leu284Arg)	Gain	20	1.09 ± 0.17[*]	12	<0.0001	0.42 ± 0.24[f]	22	<0.0001	Pathogenic
p.(Leu284Pro)	Gain	10	1.22 ± 0.14[*]	12	<0.0001	0.42 ± 0.17[f]	23	<0.0001	Pathogenic
p.(Leu284Met)	Gain	57	0.67 ± 0.22[*]	11	<0.0001	0.88 ± 0.37	24	0.9999	Pathogenic
p.(Thr287Ile)	Gain	60	0.52 ± 0.08[*]	11	<0.0001	1.17 ± 0.47	24	0.9999	Pathogenic
p.(Thr288Asn)	Gain	60	0.45 ± 0.10[*]	11	<0.0001	0.79 ± 0.32	24	0.4149	Pathogenic
p.(Leu293His)	Gain	52	0.50 ± 0.14[*]	11	<0.0001	0.87 ± 0.59	24	0.2268	Likely Pathogenic
p.(Ile300Thr)	Gain	20	0.99 ± 0.19[*]	15	<0.0001	0.35 ± 0.11[e]	22	<0.0001	Pathogenic
p.(Pro301Leu)	Loss	2 400	-1.15 ± 0.06[*]	11	<0.0001	0.46 ± 0.34[*]	23	<0.0001	Likely Pathogenic
p.(Tyr302Cys)	Loss	1 100	-0.81 ± 0.13[*]	12	<0.0001	0.64 ± 0.26	22	0.0007	Pathogenic
p.(Ala305Thr)	Gain	58	0.46 ± 0.13[*]	10	<0.0001	0.90 ± 0.33	23	0.9999	Pathogenic

p.(Ala305Val)	Gain	49	0.53 ± 0.12*	10	<0.0001	0.87 ± 0.23	24	0.9999	Pathogenic
p.(Ile306Thr)	Gain	68	0.38 ± 0.18*	10	<0.0001	0.88 ± 0.57	24	0.9999	Pathogenic
p.(Leu310Ile)	None	190	0.10 ± 0.15	17	0.1595	0.98 ± 0.57	28	0.9999	Uncertain
p.(Phe318Ser)	Loss	570	-0.44 ± 0.14*	12	<0.0001	1.06 ± 0.62	29	0.9999	Pathogenic
p.(Asn328Asp)	Loss	410	-0.25 ± 0.15*	12	<0.0001	1.07 ± 0.54	39	0.9999	Pathogenic
p.(Glu357Lys)	None	210	0.00 ± 0.13	12	0.9999	1.01 ± 0.52	29	0.9999	Likely Benign
p.(Tyr402*)	Loss		N.D.			N.D.			Likely Pathogenic
p.(Ser420Ile)	None	270	-0.02 ± 0.11	12	0.9997	0.86 ± 0.50	36	0.2084	Uncertain
p.(Arg429Gln)	None	180	0.079 ± 0.10	12	0.9020	1.12 ± 0.59	29	0.9999	Likely Benign
p.(Ser433Leu)	None	170	0.09 ± 0.13	12	0.6538	0.93 ± 0.58	29	0.9999	Uncertain
p.(Trp451fs*)	Loss		N.D.			N.D.			Likely Pathogenic

Notes:

N.D. Not determined

"Gain" is where the $\Delta logEC_{50}$ or Normalized I_{max} value has increased compared to wild-type with a p-value < 0.0001, "Loss" is where the $\Delta logEC_{50}$ or Normalized I_{max} value has decreased compared to wild-type with a p-value < 0.0001, "None" is where the p-value > 0.0001 for both tests.

p-value for a one-way ANOVA of $\Delta logEC_{50}$ values with Dunnett's post-hoc test compared to wild-type.

p-value for a non-parametric ANOVA of Normalized I_{max} values and Dunn's corrected post-hoc test compared to wild-type.

Classified according to criteria defined in (*34*). A significant change in $logEC_{50}$ in this assay was defined as PS3. No change of $logEC_{50}$ or I_{max} was not defined as benign.

[*]Considered significantly different at p < 0.0001 and categorized as gain, or loss-of-function

[e]Excess deactivation was identified

[f]Constitutive activity recorded that confounds I_{max} values

Supplementary Figure S2.

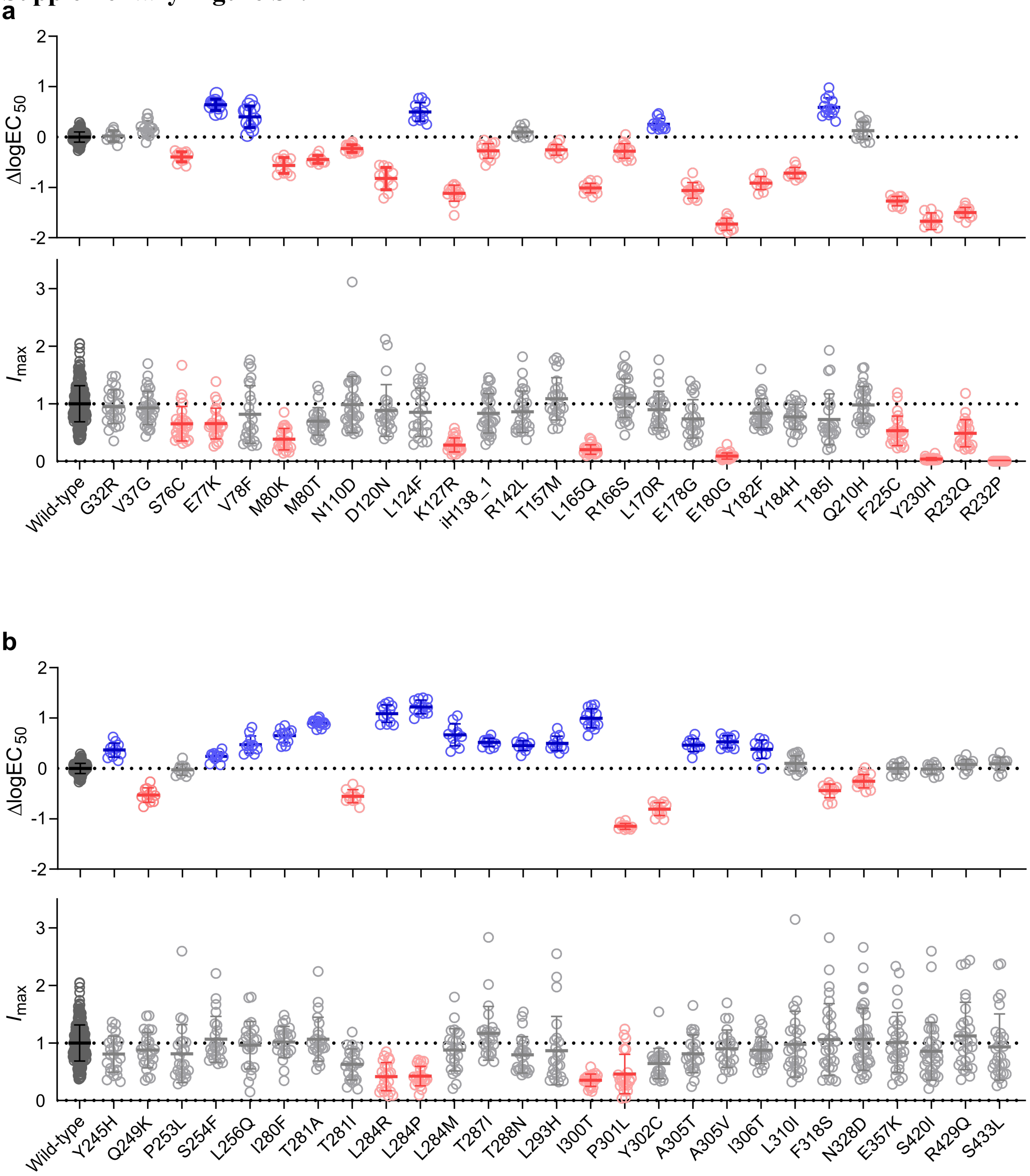

Individual $\Delta\log EC_{50}$ and I_{max} at all variants. Individual $\Delta\log EC_{50}$ (open circles, above) and I_{max} (open circles, below) values are shown with the mean ± s.d. displayed as solid lines for **a** wild-type, (Gly32Arg), p.(Val37Gly), p.(Ser76Cys), p.(Glu77Lys), p.(Val78Phe), p.(Met80Lys), p.(Met80Thr), p.(Asn110Asp), p.(Asp120Asn), p.(Leu124Phe), p.(Lys127Arg), p.(ins138_139His), p.(Arg142Leu), p.(Thr157Met), p.(Leu165Gln), p.(Arg166Ser), p.(Leu170Arg), p.(Glu178Gly), p.(Glu180Gly), p.(Tyr182Phe), p.(Tyr184His), p.(Thr185Ile), p.(Gln210His), p.(Phe225Cys), p.(Tyr230His), p.(Arg232Gln) and p.(Arg232Pro); **b** wild-type, p.(Tyr245His), p.(Gln249Lys), p.(Pro253Leu), p.(Ser254Phe), p.(Leu256Gln), p.(Ile280Phe), p.(Thr281Ala), p.(Thr281Ile), p.(Leu284Arg), p.(Leu284Pro), p.(Leu284Met), p.(Thr287Ile), p.(Thr288Asn), p.(Leu293His), p.(Ile300Thr), p.(Pro301Leu), p.(Tyr302Cys) and p.(Ala305Thr), p.(Ala305Val), p.(Ile306Thr), p.(Leu310Ile), p.(Phe318Ser), p.(Asn328Asp), p.(Glu357Lys), p.(Ser420Ile), p.(Arg429Gln) and p.(Ser433Leu). Wild-type is shown in black, gain-of-function (increased $\Delta\log EC_{50}$) are shown in blue, loss-of-function (decreased $\Delta\log EC_{50}$ and/or I_{max}) are shown in red and no change is shown in grey.

Constitutive Activity

Constitutive activity is a biophysical property of GABA$_A$ receptor variants whereby receptors are open in the absence of GABA. This confounds measurement of maximum current amplitudes by two mechanisms: (1) current from the constitutive activity is removed from measurement of maximum current amplitudes and (2) the electrical driving force can be altered by the exchange of chloride ions during the incubation period where oocytes are expressing receptors prior to recording. For example, constitutive activity was noted when voltage clamp was applied to oocytes expressing the p.(Leu284Arg) variant, which was confirmed by picrotoxin block of open receptors (Fig. S3).

Supplementary Figure S3.

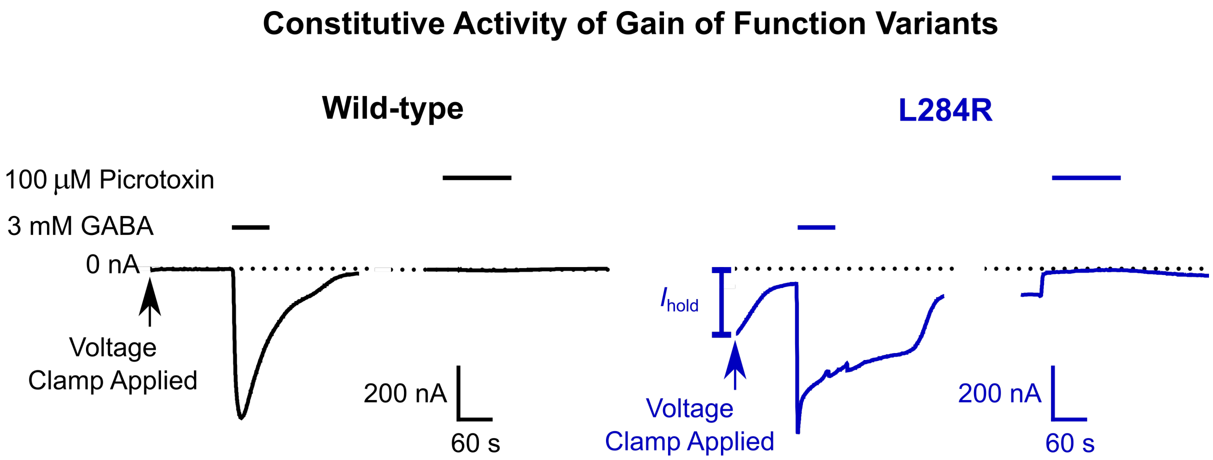

Constitutive activity of gain of function variants. Representative traces from a single two-electrode voltage clamp experiment at wild-type receptors (black) and gain-of-function β3^{L284R} (blue). Oocytes were voltage-clamped at -60 mV as indicated by the arrow, with an initial holding current (I_{hold}) that reduced over the course of a few minutes at β3^{L284R} receptors but was still less than zero (dotted line). 3 mM GABA (bars) were applied to estimate the maximum current at each oocyte. 100 µM picrotoxin (bars) was applied to demonstrate that the holding current was not non-specific leak current from the experimental set up.

Structure-Function Correlation of Variants

GABA$_A$ receptors are grouped into structural motifs, extracellular domains, transmembrane (M1-M4) and intracellular regions. The GABA-binding sites are located at the β3-α1 interfaces within the extracellular domain. Two molecules of GABA are bound to the pentamer to activate the receptor. This activation process is well described, whereby a concerted conformational sequential movements in the structural motifs that begin at the ligand-bind site and M2-M3 regions, eventually ending with a tilt of the M2 regions to open the pore (*35*). To analyse how variants located at different structural motifs altered receptor activation properties, with a view to be able to predict the functional consequences of a variant, we grouped the *GABRB3* variants based on their location on the structural motifs. These motifs included variants of amino acid residues surrounding the ligand-binding site within 10 Å of GABA, residues in the extracellular (β1-β2 and β6-β7) or transmembrane connecting loops

(M2-M3), residues within transmembrane regions M1, M2 and M3, and residues in the extracellular domain that are not in any obvious structural motif and the M3-M4 loop.

Binding Residues

GABA binds at the extracellular interface between the β3 subunit on the principal (+) and the α1 on the complementary sides. Aromatic residues on three loops of the principal side, including Y122 on loop A, Y182 on loop B, and F225 and Y230 on loop C, surround the basic amino nitrogen of GABA to make cation–π interactions with F225 and Y230 (*36, 37*). The residues E180 and Y182 on loop B are likely to form hydrogen bonds with the same amino group of GABA (*36*). Remarkably, almost all β3 amino acid residues highlighted in two cryoEM structures as essential in GABA binding also contained variants associated with epilepsy (*36, 37*).

In total, eleven variants were identified that were in close vicinity where the GABA molecule binds. These could be split into two types, four variants where the structure came within 4 Å of the GABA molecule that we define as the "inner" binding ring, and residues within 4 – 10 Å of the GABA molecule, the "outer" binding ring (Fig. 5A,B; Fig. S4).

Variants in the inner binding ring were exclusively loss-of-function, with an 8 to 53-fold decrease in GABA sensitivity (Fig. 5A,b). This is logical as residues in the inner binding ring are essential for GABA binding, making direct contacts with the GABA molecule and are highly specific for this purpose. As such, it is unsurprising that variants at these residues are loss-of-function.

Although the seven variants at the outer ring of GABA-binding residues were predominately loss-of-function (Fig. S4C,D), there was still a mix of gain-of-function variants with a 3 to 4-fold increase in GABA sensitivity ($\beta3^{L124F}$, $\beta3^{T185I}$) and loss-of-function variants with a 5 to 30-fold decrease in GABA sensitivity ($\beta3^{D120N}$, $\beta3^{K127R}$, $\beta3^{E178G}$, $\beta3^{Y184H}$, $\beta3^{R232P}$, and $\beta3^{R232Q}$). These residues are likely to be either involved in maintaining the integrity of the GABA-binding site or at the early stages of the activation pathway.

Supplementary Figure S4.

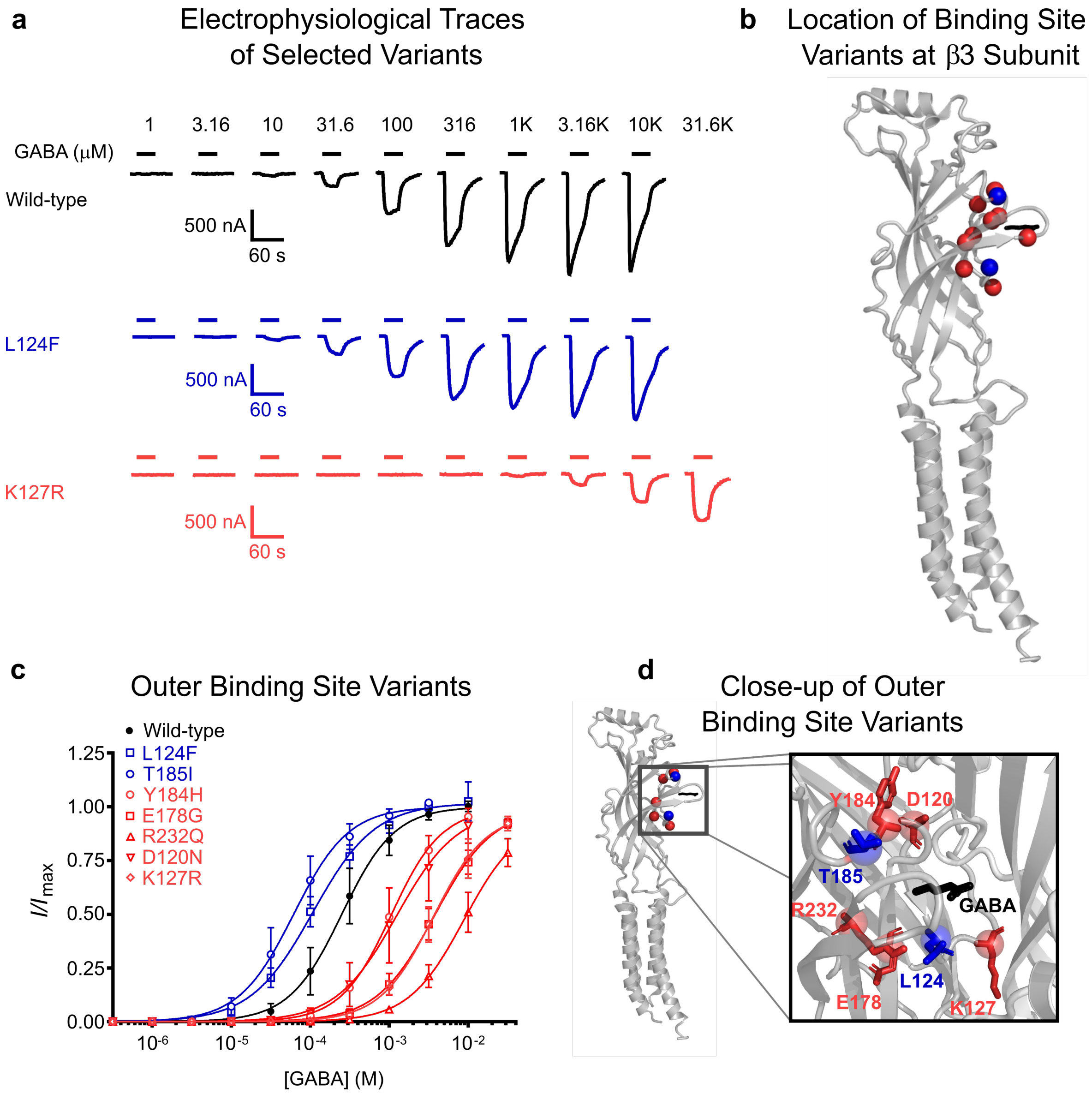

Function of variants mapped to the GABA-binding site. a. Representative traces from a single two-electrode voltage clamp experiment at wild-type receptors (black), the gain-of-function $\beta3^{L124F}$ (blue) and loss-of-function $\beta3^{K127R}$ (red). Increasing concentrations of GABA (bars) were applied to construct a concentration-response curve to GABA. **b.** Structure of the β3 subunit with the backbone coloured in grey. Red, and blue spheres indicate residues at the binding site containing variants with a decrease or increase in GABA potency, respectively **c.** Concentration-response curves for variants at the outer binding site. Dots represent mean ± s.d. and lines the fitted Hill equation. Wild-type concentration-response curves run on the same days as the variants are shown in black. Red indicates loss-of-function and blue indicates gain-of-function. n = 35, 12, 13, 13, 11, 14, 11, independent experiments at wild-type, p.(Leu124Phe), p.(Thr185Ile), p.(Tyr184His), p.(Glu178Gly), p.(Arg232Gln), p.(Asp120Asn), p.(Lys127Arg). **d**. Close-up view of the structures of the outer binding site displaying the amino acid sidechains of the residues containing variants in sticks. Red indicates loss-of-function, blue indicates gain-of-function variants and GABA is in black. Source data are provided as a Source Data file.

Coupling Regions

As the energy from GABA binding at the extracellular domain is conducted toward the channel pore, motifs at the interface of the extracellular domain and transmembrane domains shift conformation. These motifs include the β1-β2, β6-β7 and pre-M1 extracellular coupling loops and the extracellular M2-M3 loop that connects the two transmembrane regions. These amino acid residues are more likely involved in stabilizing either an intermediate or open state of the receptor, hence gain-of-function residues would be expected to feature more prominently. The coupling region between the extracellular and transmembrane domains is intimately involved in stabilizing intermediate states of the receptor, and unsurprisingly harbours a cluster of epilepsy-associated variants, fourteen in all (Fig. 5C,D; Fig. S5).

Residues in the extracellular coupling loops clearly played a significant role in channel gating, with a mix of gain and loss-of-function variants found in this region. Variants found in the β1-β2 extracellular coupling loop were a mix of gain- ($β3^{E77K}$, $β3^{V78F}$) and loss- of-function ($β3^{S76C}$, $β3^{M80T}$, $β3^{M80K}$) variants with a 2.5 to 4.4-fold increase or a 2.5 to 3.6-fold decrease in GABA sensitivity, respectively (Fig. 5C). Meanwhile, variants found in the β6-β7 coupling loop were also a mix of gain- ($β3^{L170R}$) and loss-of-function ($β3^{L165Q}$, $β3^{R166S}$) with a 1.8-fold increase or a 2.0 to 10-fold decrease in GABA sensitivity, respectively (Fig. S5).

Finally, the M2-M3 coupling loop that couples the transmembrane regions to the extracellular domain makes a distinct, early movement in the activation process. As expected, gain-of-function variants ($β3^{I300T}$, $β3^{A305T}$, $β3^{A305V}$ and $β3^{I306T}$) were more prevalent than loss-of-function ($β3^{P301L}$ and $β3^{Y302C}$) at this region, with a 2.4 to 10-fold increase or a 6.4 to 14-fold decrease in GABA sensitivity, respectively (Fig. 5D).

Supplementary Figure S5.

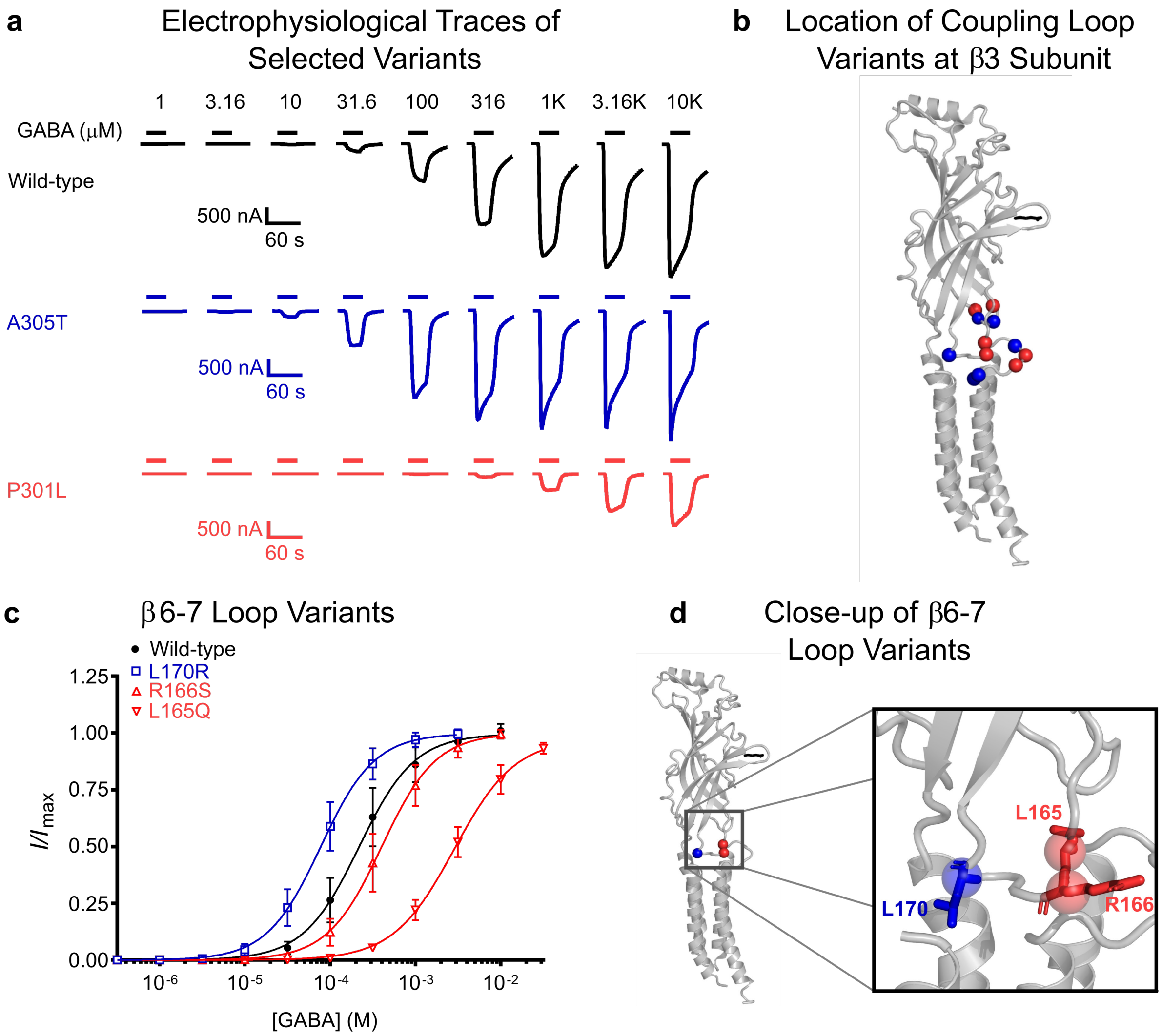

Function of variants mapped to the coupling regions. a. Representative traces from a single two-electrode voltage clamp experiment at wild-type receptors (black), the gain-of-function $\beta3^{A305T}$ (blue) and loss-of-function $\beta3^{P301L}$ (red) receptors. Increasing concentrations of GABA (bars) were applied to construct a concentration-response curve. **b.** Structure of the β3 subunit with the backbone coloured in grey. Red, and blue spheres indicate residues containing variants in the coupling loops with a decrease or increase in GABA potency, respectively **c.** Concentration-response curves for variants at the β6-β7 coupling loop. Dots represent mean ± s.d. and lines the fitted Hill equation. Wild-type concentration-response curves run on the same days as the variants are shown in black. Red indicates loss-of-function and blue indicates gain-of-function. n = 41, 14, 15 and 12 independent experiments at wild-type, p.(Leu165Gln), p.(Arg166Ser), p.(Leu170Arg). **d.** Close-up of the structures of the β6-β7 coupling loop displaying the amino acid sidechains of the residues containing variants in sticks. Red indicates loss-of-function, blue indicates gain-of-function. Source data are provided as a Source Data file.

Transmembrane Regions

The transmembrane regions anchor the protein within the lipid membrane of the cell and, most importantly, the second transmembrane domain (M2) surrounds the ion channel pore. Seventeen variants were located in the transmembrane regions (Fig. S6) and nine of these were located in the M2 region. The heavy concentration of variants within the M2 region is a consequence both of the importance of this region in the transition of the receptor from close to open, and that amino acid sidechains of the M2 region surrounds the chloride channel pore (*31, 35-37*)(Fig. 5E).

Of the variants located in the transmembrane region of the receptor, there was a trend toward gain in the first transmembrane (M1) region, ($\beta3^{Y245H}$, $\beta3^{S254F}$ and $\beta3^{L256Q}$) than loss-of-function ($\beta3^{Q249K}$) variants with a 1.7 to 3.0-fold increase or a 3.4-fold decrease in GABA sensitivity, respectively. The $\beta3^{P253L}$ variant in the M1 had no change in GABA sensitivity, however increased rates of deactivation were identified in individual traces.

Strikingly, variants in the M2 region were almost exclusively gain-of-function ($\beta3^{I280F}$, $\beta3^{T281A}$, $\beta3^{L284R}$, $\beta3^{L284P}$, $\beta3^{L284M}$, $\beta3^{T287I}$, $\beta3^{T288N}$, and $\beta3^{L293H}$) with a 2.8 to 17-fold increase in GABA sensitivity. Only the $\beta3^{T281I}$ variant reduced the GABA sensitivity by 3.6-fold. Curiously, variants at the T281 residue can be either loss or gain-of-function. The finding that these variants are mainly gain-of-function correlates strongly with the role of the M2 region in channel gating.

In the third transmembrane (M3) region, there were two loss-of-function ($\beta3^{F318S}$ and $\beta3^{N328D}$) variants with a 1.8 to 2.8-fold increase in GABA sensitivity, and one with no functional change ($\beta3^{L310I}$) (Fig. S6, Table S2).

Overall, there is a clear trend for variants in residues contributing early in the channel activation pathway to be loss of function, and those contributing later to be a gain, but the trend is not clear enough to make functional predictions based on the variant alone.

Supplementary Figure S6.

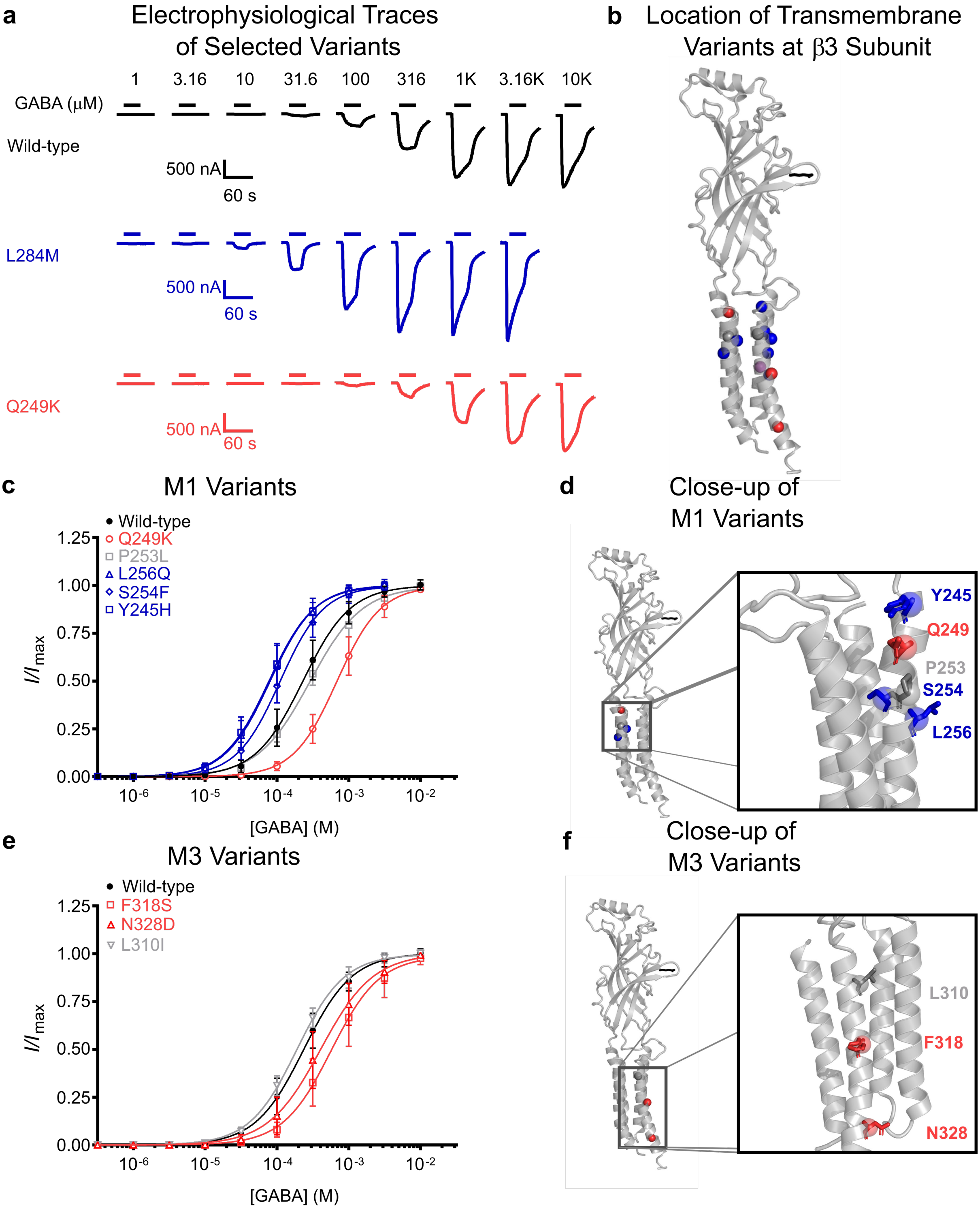

Function of variants mapped to transmembrane regions. a. Representative traces from a single two-electrode voltage clamp experiment at wild-type receptors (black), the gain-of-function $\beta3^{L284M}$ (blue) and loss-of-function $\beta3^{Q249K}$ (red) receptors. Increasing concentrations of GABA (bars) were applied to construct a concentration-response curve to GABA. **b.** Structure of the β3 subunit with the backbone coloured in grey. Red, and blue spheres indicate residues containing variants in the transmembrane regions with a decrease or increase in GABA potency, respectively. Grey spheres indicate no functional change and purple indicates residues with both gain and loss-of-function variant. **c and e.** Concentration-response curves for variants at the **c** M1 and **e** M3 transmembrane helices. Dots represent mean ± s.d. and lines the fitted Hill equation. *Legend continues next page…*

Legend Figure S6 continued:
Wild-type concentration-response curves run on the same days as the variants are shown in black. Red indicates loss-of-function and blue indicates gain-of-function. n = 47, 12, 11, 12, 11 and 11 independent experiments at wild-type, p.(Tyr245His), p.(Gln249Lys), p.(Pro253Leu), p.(Ser254Phe), p.(Leu256Gln). n = 34, 12, 12 and 12 independent experiments at wild-type, p.(Leu310Ile), p.(Phe318Ser), p.(Asn328Asp). **d and f**. Close-up view of the structures of the **d** M1 and **f** M3 transmembrane helices displaying the amino acid sidechains of the residues containing variants in sticks. Red indicates loss-of-function, blue indicates gain-of-function, grey indicates no functional change and purple indicates residues with both gain and loss-of-function variant. Source data are provided as a Source Data file.

Other Extracellular and M3-M4 Variants

There were several other variants in regions not thought to be involved in the binding site or activation pathway. Seven of these variants were found in the extracellular domain. Of these, $\beta3^{G32R}$, $\beta3^{V37G}$, $\beta3^{R142L}$ and $\beta3^{Q210H}$ did not display any functional change, while $\beta3^{N110D}$, $\beta3^{T157M}$ and $\beta3^{insHis138\_1}$ were loss-of-function variants with a 1.7 to 1.9-fold decrease in GABA sensitivity. A further three variants were within the M3-M4 loop, $\beta3^{E357K}$, $\beta3^{R429Q}$, $\beta3^{S433L}$ all of which did not display any functional change ($\Delta logEC_{50}$ = 0.00 – 0.11) (Fig. S7, Table S2).

Supplementary Figure S7.

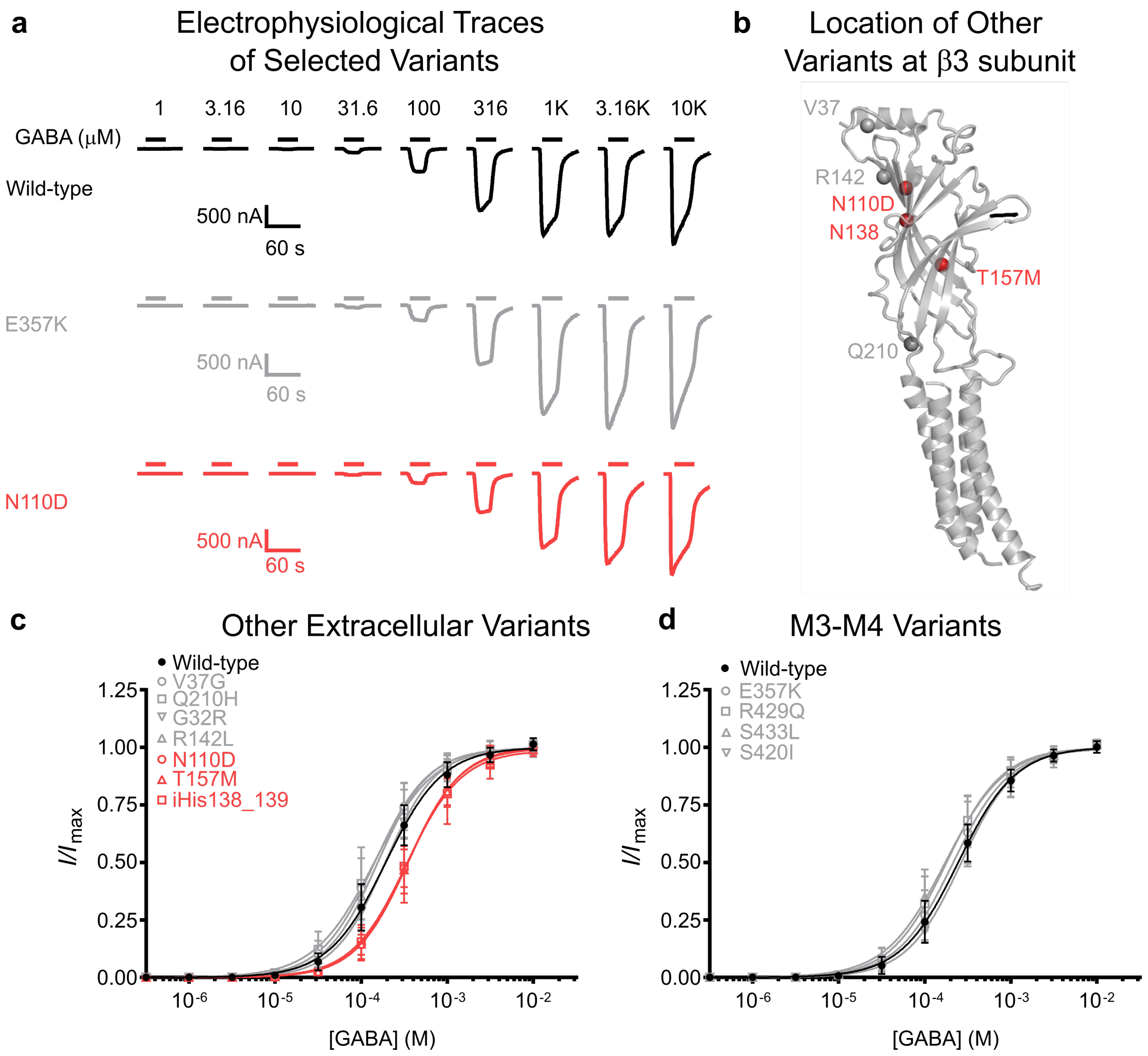

Function of variants at unassigned extracellular and intracellular regions. a. Representative traces from a single two-electrode voltage clamp experiment at wild-type receptors (black), the no functional change $\beta3^{E357K}$ (grey) and loss-of-function $\beta3^{N110D}$ (red). Different concentrations of GABA (bars) were applied to construct a concentration-response curve to GABA. **b.** Structure of the β3 subunit with the backbone coloured in grey. Red spheres indicate residues with a decrease in GABA potency, grey spheres indicate no functional change and purple indicates residues with both gain and loss-of-function variant. **c-d.** Concentration-response curves for variants at the **c** extracellular and **d** M3-M4. Dots represent mean ± s.d. and lines the fitted Hill equation (line). Wild-type concentration-response curves run on the same days as the variants are shown in black. Red indicates loss-of-function and grey indicates no functional change. n = 41, 10, 15, 15, 15, 10, 11 and 14 independent experiments at wild-type, p.(Gly32Arg), p.(Val37Gly), p.(Asn110Asp), p.(ins138_139His), p.(Arg142Leu), p.(Thr157Met), p.(Gln210His). n = 30, 12, 12, 12 and 12 independent experiments at wild-type, p.(Glu357Lys), p.(Ser420Ile), p.(Arg429Gln), p.(Ser433Leu). Source data are provided as a Source Data file.

Section 6: Detailed seizure types of gain- and loss-of-function variants

Seizure Types

Seizure types were compared between patients harbouring a gain- or loss-of-function variants. Both the seizure types reported at onset and seizure types reported at follow-up were analysed separately. Focal, focal to bilateral and myoclonic seizures are the most frequently reported in patients with gain-of-function variants while febrile and bilateral to tonic-clonic seizures are most frequently reported in patients with loss-of function variants (Fig. S8).

Supplementary Figure S8.

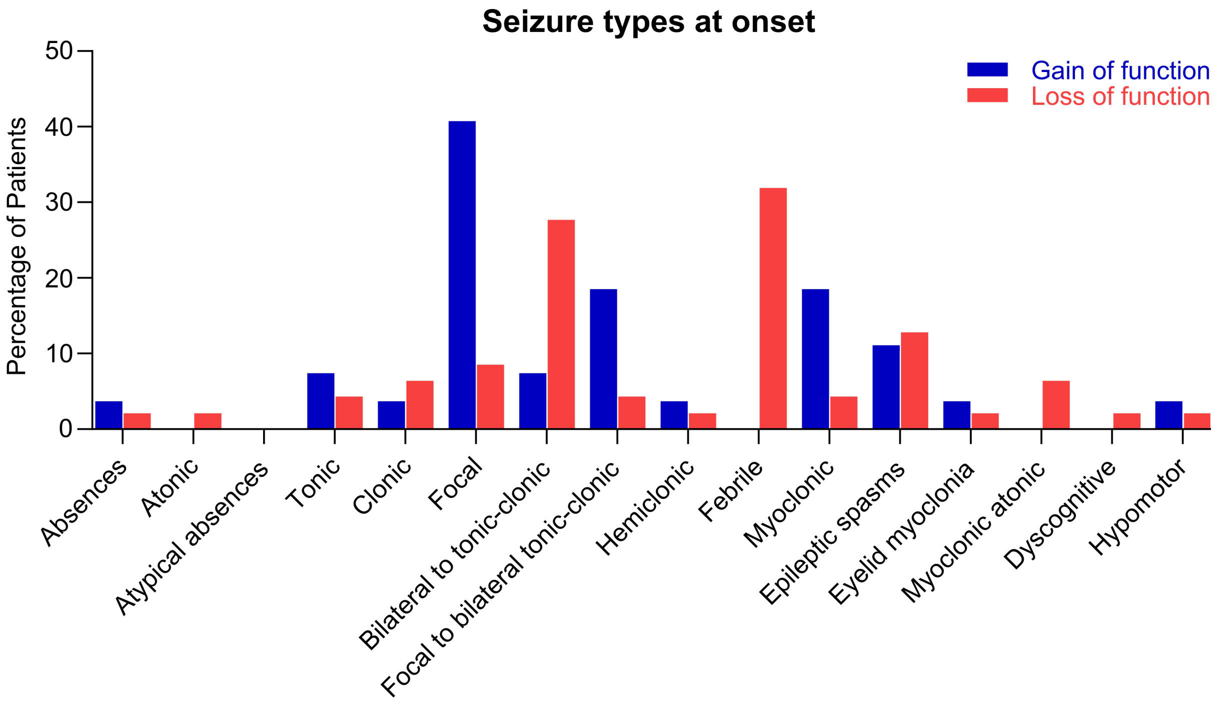

Seizure types at onset. Bar graph showing the percentage of patients recorded as presenting with specific seizure types at onset of epileptic disorders. Blue bars represent patients with gain-of-function variants and red bars represent loss-of-function variants. Source data are provided in Supplementary Table S1.

Seizure Types at follow-up

Although focal seizures remained the most frequently reported in gain-of-function patients, tonic seizures were the second most frequently seizure type reported on follow-up. Febrile seizures at loss-of function variants were less frequently reported at follow-up than at onset, while absence, atypical absence, atonic, tonic and myoclonic seizures were more frequently reported at follow-up. This is indicative of loss-of-function patients following a similar generalized epilepsy with febrile seizures plus $GEFS^+$ spectrum of *SCN1A* variants, where febrile seizures are initially reported that develop into a variety of more severe seizure types (Fig. S9).

Supplementary Figure S9.

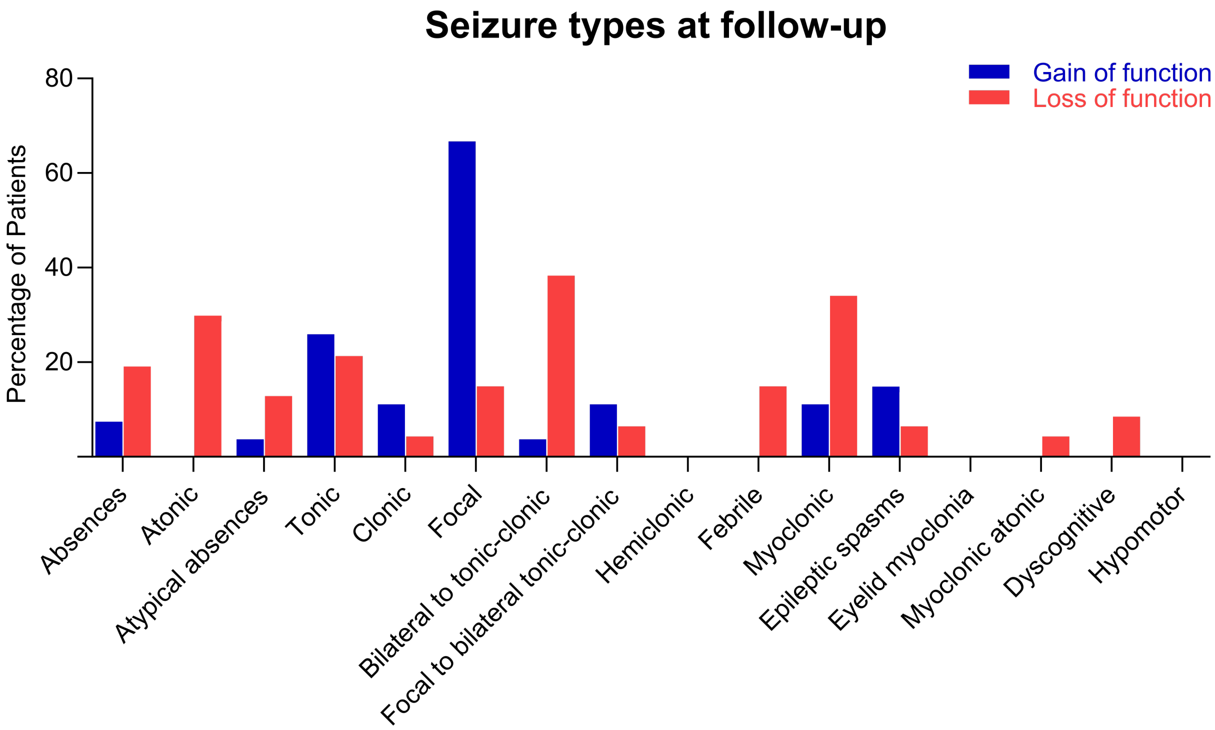

Seizure types at follow up. Bar graph showing the percentage of patients recorded as presenting with specific seizure types at follow-up of epileptic disorders. Blue bars represent patients with gain-of-function variants and red bars represent loss-of-function variants. Source data are provided in Supplementary Table S1.

Section 7: Detailed analysis of intellectual disability at gain- and loss-of-function variants

A range of intellectual disability severity was reported at *GABRB3* variants from normal to severe. However, when genotype/phenotype correlations were performed, severe intellectual disability was strongly associated with gain-of-function variants. This demonstrates that the severity of intellectual disability is strongly correlated with the type of variant (Fig. S10, Table S3).

Supplementary Figure S10.

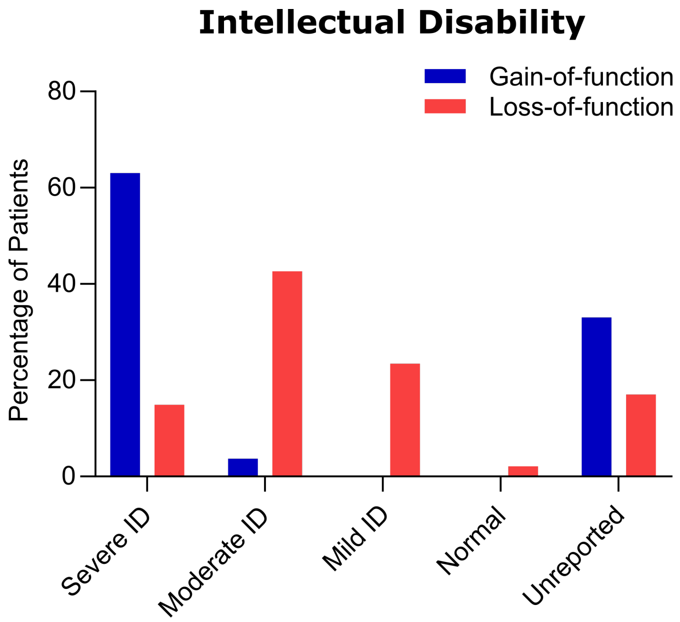

Severity of intellectual disability at gain- and loss-of-function variants. Bar graph showing the percentage of patients recorded as presenting with intellectual disabilities of different severity for gain-of-function (blue bars) and loss-of function (red bars) patients. A strong association between gain-of-function patients and severe intellectual disability was found. Source data are provided in Supplementary Table S1.

Supplementary Table S3.

Summary of Intellectual Disabilities at all *GABRB3* variants.

Intellectual Disability	Gain-of-function	Loss-of-function	No functional change
Severe	17	7	2
Moderate	1	20	3
Mild	0	11	2
Normal	0	1	4
Unreported	9	8	0

Section 8: Detailed comorbidities at gain- and loss-of-function variants

Odds Ratio Analysis

An odds ratio analysis was performed for gain- and loss-of-function variants with a variety of comorbidities including neuropsychiatric and movement disorders, speech issues, sleep disturbances, pyramidal and extrapyramidal signs, scoliosis, digestive issues, hypotonia, microcephaly and vision impairment (Fig. S11). Digestive issues, hypotonia, microcephaly and vision impairment were enriched at the gain-of-function variants. Low numbers of reported co-morbidities precluded reliable statistical analysis at other co-morbidities.

Supplementary Figure S11.

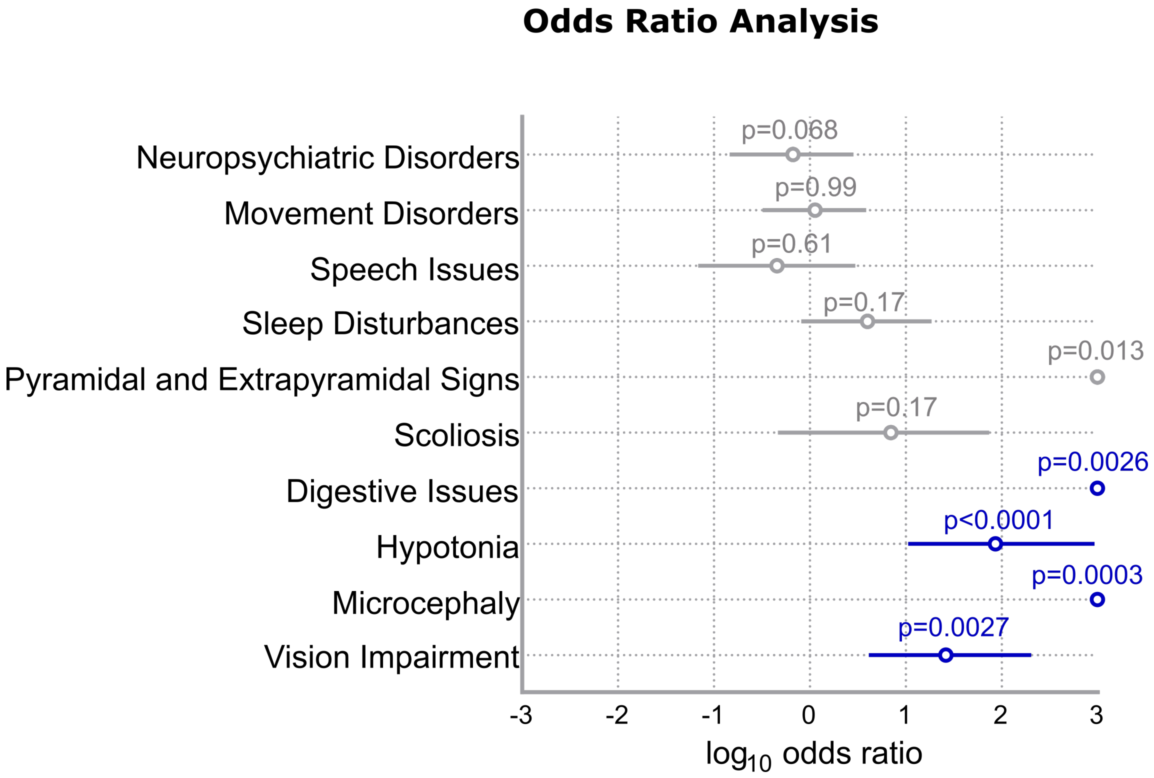

Odds ratio analysis of co-morbidities at gain- and loss-of-function variants. Odds ratio of clinical outcomes in gain-of-function vs. loss-of-function variants with the centre circle showing the $\log_{10}$ odds ratio and 95 % confidence interval (dots with bars) at each co-morbidity with the p-value displayed from Fischer's exact test, two-sided ($p = 0.68$, 0.99, 0.61, 0.17, 0.013, 0.17, 0.0026, 3.5×10^{-7}, 3×10^{-4} and 0.0027). Co-morbidities that are significantly enriched ($p < 0.01$) in patients with gain-of-function variants (blue) and no significant difference (grey) are shown. For each indication, the total number of patients reporting were n = 27, 27, 7, 9, 8, 8, 8, 16, 11 and 7 gain-of-function patients, and n = 47, 47, 27, 24, 22, 22, 22, 27, 24 and 23 loss-of-function patients for neuropsychiatric disorders, movement disorders, speech issues, sleep disturbances, pyramidal and extrapyramidal signs, scoliosis, digestive issues, hypotonia, microcephaly and vision impairment respectively. Source data are provided in Supplementary Table S1.

Cluster Analysis

We ran both a two-step model and a neural network (multilayer perceptron) in SPSS statistics program analysing age of onset, severity of intellectual disability and all seizure types. The two-step model predicted two clusters with 100% accuracy for the gain and loss-of-function variants. The multilayer perceptron was 100% accurate in picking GOF variants into a cluster, and 94% for loss-of-function.

To test the validity of our flow chart we again ran both a two-step model and a neural network (multilayer perceptron) in SPSS statistics program analysing the category of age of onset (i.e greater or less than 6 months), hypotonia, focal and febrile seizures. The analysis was 100% accurate in picking GOF variants and 92% accurate in picking LOF variants. The multilayer perceptron was 100% accurate for GOF variants and 89 and 100% accurate in picking LOF variants (testing and training respectively). The flowchart accounts for these errors in LOF diagnosis for having ambiguous pathways where the functional change of the variant ought to be tested. In all cases, the results of the cluster analysis were no different to the more rigorous analysis performed in the paper.

Movement Disorders

Movement disorders including dystonia, choreoathetosis, ataxia, dyskinesia, paroxysmal dyskinesia and spasticity were recorded with reports of similar numbers in patients with gain- and loss-of-function variants (Table S4).

Supplementary Table S4.

Summary of Movement Disorders at all *GABRB3* variants.

	GOF	LOF	No functional change
Total	4/27 (15%)	6/47 (13%)	1/11 (9%)
Dystonia	2	0	0
Choreoathetosis	1	0	0
Ataxia	0	6	1
Dyskinesia	2	0	0
Paroxysmal Dyskinesia	0	0	0
Spasticity	2	0	0

Neuropsychiatric Disorders

Neuropsychiatric disorders including autism, autistic features, ADHD, stereotypical behaviour, aggression, anxiety and other behavioural disorders were recorded. A greater proportion of neuropsychiatric disorders were recorded for the loss-of-function variants, but they were not significantly enriched. It is also possible that the severe intellectual disabilities in patients with gain-of-function variants masks the clinical presentation of neuropsychiatric disorders (Table S5).

Supplementary Table S5.

Summary of neuropsychiatric disorders at all *GABRB3* variants.

	GOF	LOF	No functional change
Autism	0	6	1
Autistic Features	1	8	2
ADHD	0	7	0
Stereotypical Behaviour	1	5	0
Aggression	0	5	1
Anxiety	0	2	1
Other	3	4	1
None	3	9	5

Other Comorbidities

The remaining comorbidities including hypotonia, microcephaly, speech issues, vision impairment, scoliosis, pyramidal and extrapyramidal signs, sleep disturbance and digestive issues were recorded. Hypotonia, microcephaly, vision impairment and digestive issues were all significantly enriched at gain-of-function patients, while other comorbidities were not significantly different between the two patient groups (Table S6).

Supplementary Table S6.

Summary of other comorbidities at all *GABRB3* variants.

	GOF	LOF	No change	OR [95% CI]	p-value
Hypotonia	15/16 (94 %)	4/27 (15 %)	2/6 (33 %)	86 [11-933]	< 0.0001
Microcephaly	6/11 (55 %)	0/24 (0 %)	1/5 (20 %)	ND	0.0003
Speech Issues	5/7 (71 %)	22/27 (81 %)	4/7 (57 %)	0.57 [0.10-3.6]	0.6148
Vision Impairment	5/7 (71 %)	2/23 (9 %)	1/5 (20 %)	26 [3.4-170]	0.0027
Scoliosis	2/8 (22 %)	1/22 (5 %)	0/5 (0 %)	7 [0.67-105]	0.1655
Pyramidal and Extrapyramidal Signs	3/8 (38 %)	0/22 (0 %)	0/6 (0 %)	ND	0.0138
Sleep Disturbance	4/9 (44 %)	4/24 (17 %)	1/6 (17 %)	4 [0.86-20]	0.1695
Digestive Issues	4/8 (50 %)	0/22 (0 %)	1/6 (17 %)	ND	0.0007

Notes: Odds ratio and p-value are comparisons between GOF and LOF patients.
P-value displayed from Fischer's exact test, two-sided (p = 0.68, 0.99, 0.61, 0.17, 0.013, 0.17, 0.0026, 3.5×10^{-7}, 3×10^{-4} and 0.0027)

Section 9: Drug reactions at gain- and loss-of-function variants

The reactions of patients with both gain- and loss-of-function patients to different anti-seizure medications were recorded. Statistical evaluation of individual drugs was not possible due to low numbers, however when separated into mechanism of action, clear trends in responses to different drug classes were evident that will assist neurologists in the management of the two groups of patients.

Sodium Channel Inhibitors

Usage of sodium channel blockers including carbamazepine, oxcarbazepine, phenytoin, lacosamide and lamotrigine were reported in patients with both loss- and gain-of-function variants (Table S7). These drugs performed poorly in patients with loss-of-function variants, with only two cases where lamotrigine resulted in seizure freedom (including one in combination with clobazam) and one case of oxcarbazepine reducing seizure frequency in a total of 12 patients. In contrast, there were ten cases where sodium channel blockers had no effect, and 4 cases where seizures were worsened. This is a similar outcome for patients with GEFS+ or Dravet syndrome caused by *SCN1A* loss-of-function variants. In patients with gain-of-function *GABRB3* variants, one case of seizure freedom with carbamazepine and topiramate was recorded along with five cases that displayed no effect.

Supplementary Table S7.

Responses to sodium channel inhibitors in patients with gain and loss-of-function *GABRB3* variants.

	LOF				GOF			
	Seizure Free	Seizure Reduction	No Effect	Worsening/ Adverse	Seizure Free	Seizure Reduction	No Effect	Worsening/ Adverse
Carbamazepine	0	0	3	1	1	0	1	0
Oxcarbazepine	0	1	1	1	0	0	2	0
Phenytoin	0	0	3	1	0	0	1	0
Lacosamide	0	0	1	0	0	0	1	0
Lamotrigine	2	0	2	1	0	0	0	0

GABA enhancers

Drugs that enhance GABAergic activity, either directly, or by inhibiting GABA transport or breakdown of GABA were reported in patients with both loss- and gain-of-function variants (Table S8). These drugs performed well for patients with loss-of-function variants, with six individual reported cases of seizure freedom out of 16 patients. This is an outcome similar to patients with *SCN1A* loss-of-function variants. In contrast, s there were only two instances where GABA enhancers reduced the seizure frequency out of nine patients with gain-of-function variants, eight instances where they had no effect and caused adverse effects in three patients.

Supplementary Table S8.

Responses to GABAergic enhancers in patients with gain and loss-of-function *GABRB3* variants

	LOF				GOF			
	Seizure Free	Seizure Reduction	No Effect	Worsening/ Adverse	Seizure Free	Seizure Reduction	No Effect	Worsening/ Adverse
Clobazam	3	1	4	0	0	0	2	0
Clonazepam	1	1	2	0	0	1	2	0
Nitrazepam	1	0	0	0	0	0	0	0
Phenobarbital	2	0	3	0	0	0	3	1
Stiripentol	2	0	0	0	0	0	0	0
Vigabatrin	0	1	0	1	0	0	1	2

Carbonic anhydrase inhibitors

Drugs that inhibit carbonic anhydrase, including sulthiame and topiramate were reported to be prescribed to patients with both loss and gain-of-function variants (Table S9). These drugs performed well in patients with loss-of-function variants, with reduction of seizure frequency in five out of eight individuals and this included two patients with seizure freedom (one topiramate and valproate, another topiramate with levetiracetam and valproate). In six patients with gain-of-function variants, the effect was less clear with one case of topiramate (with carbamazepine) leading to seizure freedom, but three cases with no effect and one case of seizures worsening.

Supplementary Table S9.

Responses to Carbonic Anhydrase Inhibitors in patients with Gain- and Loss-of-Function *GABRB3* variants

	LOF				GOF			
	Seizure Free	Seizure Reduction	No Effect	Worsening	Seizure Free	Seizure Reduction	No Effect	Worsening/ Adverse
Sultiame	0	1	0	0	0	0	1	0
Topiramate	2	2	3	0	1	1	2	1

Glutamate Receptor/ Calcium Channel Inhibition and Potassium Channel Activator

There was only one report of these classes of drugs, with no effect at a single patient with a gain-of-function variant (Table S10).

Supplementary Table S10.

Responses to glutamate receptor and calcium channel inhibitors, and potassium channel activators in patients with gain- and loss-of-function *GABRB3* variants

	LOF				GOF			
	Seizure Free	Seizure Reduction	No Effect	Worsening	Seizure Free	Seizure Reduction	No Effect	Worsening/ Adverse
Ethosuximide	0	0	0	0	0	0	0	0
Perampanel	0	0	0	0	0	0	0	0
Gabapentin	0	0	0	0	0	0	1	0
Retigabine	0	0	0	0	0	0	0	0

Other anti-epileptic drug classes with unknown or mixed actions

Levetiracetam and valproate were also relatively effective in patients with loss-of-function variants, with eight individual cases of seizure freedom, four of which the two drugs were used in combination (Table S11). However, there were 11 instances where these drugs had no effect and one case that valproate worsened seizures. In contrast, only one instance where levetiracetam reduced seizure frequency in patients of gain-of-function variants. Steroids were effective in two patients with loss-of-function variants (one in combination with valproate), while a single case of the ketogenic diet resulted in seizure freedom in a patient with a gain-of-function variant.

Supplementary Table S11.

Responses to other antiepileptic drugs in patients with gain- and loss-of-function *GABRB3* variants

	LOF				GOF			
	Seizure Free	Seizure Reduction	No Effect	Worsening/ Adverse	Seizure Free	Seizure Reduction	No Effect	Worsening/ Adverse
Levetiracetam	3	1	6	0	0	1	7	1
Valproate	8	4	5	1	0	0	5	1
Zonisamide	1	0	1	0	0	0	1	0
Steroids	2	0	1	0	0	0	1	0
Ketogenic Diet	0	0	1	0	1	0	3	0
Vagal nerve stimulation	0	0	0	0	0	0	0	0
Cannabidiol	0	0	0	0	0	0	0	0

Section 10. Opposite molecular and clinical phenotypes of variants at the T281 residue.

Curiously, variants at the $\beta3^{T281}$ residue displayed either loss- or gain-of-function variants, where the $\beta3^{T281A}$ variant increased GABA sensitivity by 8-fold and the $\beta3^{T281I}$ variant reduced GABA sensitivity by 3.6-fold (Fig. S12). Neither variant altered the maximum currents. These two variants exemplified the distinction between gain- and loss-of-function variants, with the $\beta3^{T281A}$ patient having a lower age of seizure onset, focal seizures and no response to drug treatment, while the $\beta3^{T281I}$ patient had an age of onset of seven months, febrile seizures and was seizure free after treatment with a combination of GABA enhancers and sodium valproate.

Supplementary Figure S12.

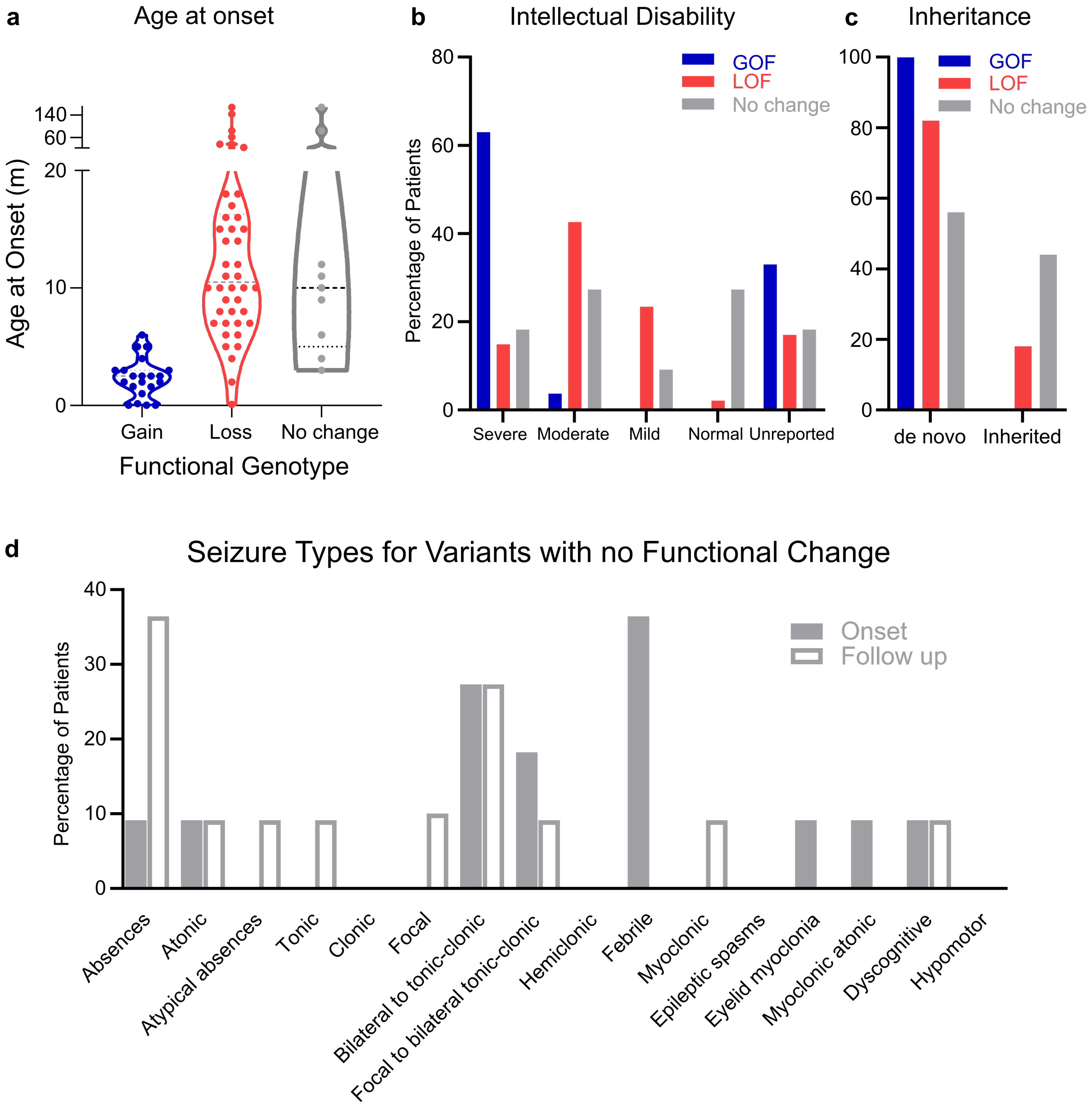

Opposite molecular and clinical phenotypes of variants at the T281 residue. a. Representative data from a single two-electrode voltage clamp experiment at wild-type receptors (black), the gain-of-function p.(Thr281Ala) (blue) and loss-of-function p.(Thr281Ile) (red) $GABA_AR$. Increasing concentrations of GABA (bars) were applied to construct a concentration-response curve to GABA. **b.** Maximum currents of wild-type (black, n = 48

independent experiments), p.(Thr281Ala) (blue, n = 24) and p.(Thr281Ile) (red, n = 24) variants. **c.** Concentration-response curves for wild-type receptors (black, n = 57 independent experiments)), the gain-of-function p.(Thr281Ala) (blue, n = 14) and loss-of-function p.(Thr281Ile) (red, n = 11) . Dots represent mean ± s.d. and lines the fitted Hill equation. Wild-type concentration-response curves run on the same days as the variants are shown in black. **d.** Selected clinical features of the p.(Thr281Ala) (blue) and p.(Thr281Ile) (red) variants. Source data are provided as a Source Data file.

Section 11: Extended results and discussion of variants with no functional change

For ten of the tested variants (11 patients), we were unable to determine whether they were loss- or gain-of-function in our electrophysiological assay. The low numbers of variants that are in this group of no functional change prevented any meaningful statistical analysis of the patient cohort. Patients harbouring a variant with no functional change presented with seizures at a median age 10 months [CI:4-84], n = 9). A greater proportion of the patients presented with no intellectual disability and a high percentage of the variants were inherited rather than *de novo*. Febrile and generalized tonic-clonic seizures were the main seizure types (Fig. S13).

Supplementary Figure S13.

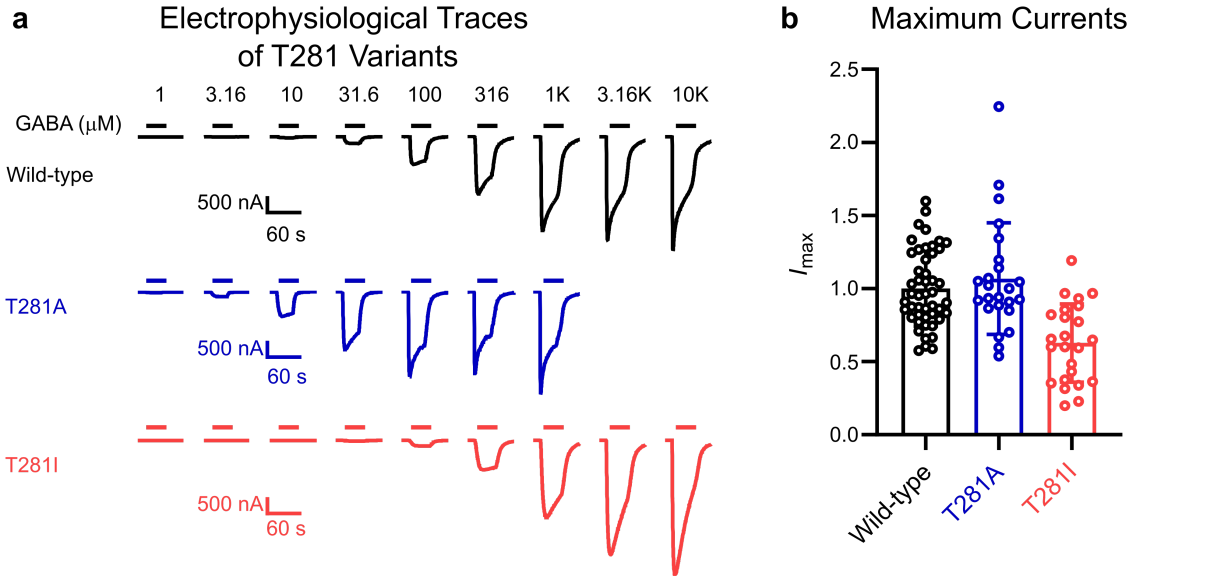

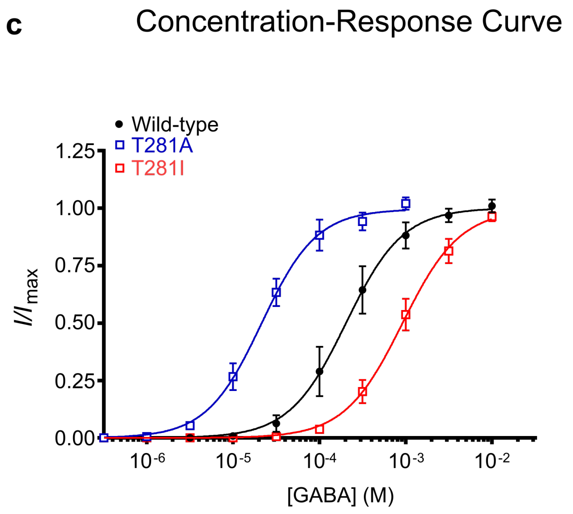

d Clinical Phenotype of T281 Variants

Variant	T281A	T281I
Type	Gain	Loss
Age at Onset (mo)	2	7
Seizure Types at Onset	Absence, Focal	Febrile
Seizure Freedom	Nil	Phenobarbital Valproate Stiripentol
No Effect	Carbamazepine Levetiracetam Phenobarbital Ketogenic Diet	Nil
Worsening	Topiramate	Nil

Clinical Description of variants with no functional change. a. Violin plot of the age of onset for patients with no functional change (grey dots, n = 9), loss-of-function (red, n = 43) and gain-of-function (blue, n = 21) variants for comparison. **b** and **c.** Bar Graph of the percentage of patients with different severities of **b** intellectual disabilities and **c** types of inheritance at variants with no functional change (grey bars), loss-of-function (red bars) and gain-of-function (blue bars). **d**. Fraction of patients with variants of no functional change for different seizure types at onset (filled bars) and follow-up (open bars). Source data are provided in Supplementary Table S1.

Section 12. Increased deactivation kinetics of the p.(Pro253Leu) variant.

On visual inspection, the traces of the receptors containing the p.(Pro253Leu) variant had sharper currents with faster deactivation times compared to wild-type (Fig. S14). We formally compared the 3 mM GABA traces to wild-type by fitting the current decay to an exponential curve $I=(I_0\text{-Plateau})e^{-kt}$ + Plateau and compared the deactivation rate constant (k). The deactivation rate constant was significantly increased from 0.021 ± 0.011 s^{-1} (mean ± s.d.) at wild-type receptors to 0.039 ± 0.016 s^{-1} at receptors containing the p.(Pro253Leu) variant ($p < 0.0005$, Mann-Whitney U-test).

Supplementary Figure S14.

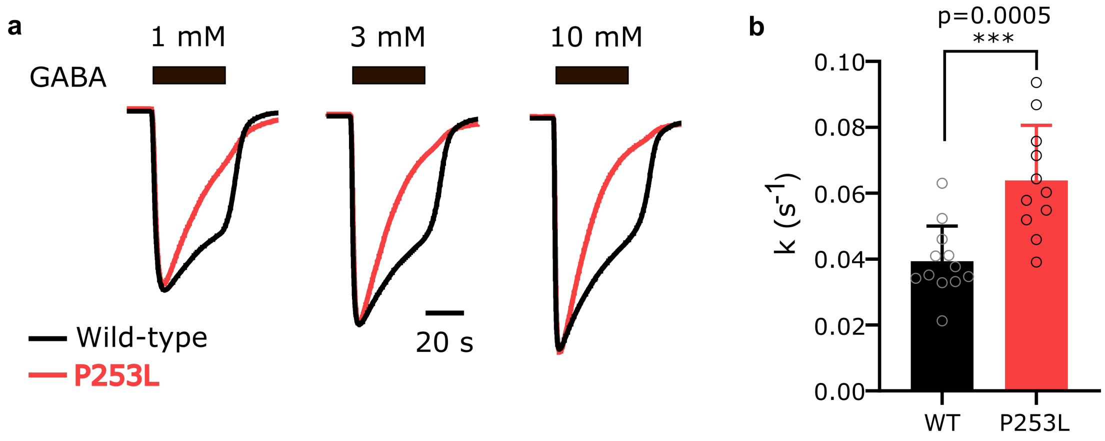

Increased deactivation rates of β3^{P253L} receptors. a. Representative traces of wild-type (black) and p.(Pro253Leu) (red) receptors with 1, 3 or 10 mM GABA applied on the same electrophysiological set up on the same day. Black bars indicate time of GABA application. For clarity, traces were all normalized to the maximum current of the respective 3 mM GABA application. **b.** Deactivation rate constants (k) were determined at each receptor when experiments were run on the same day. The bar graph displays k values for wild-type (black, n = 12) and p.(Pro253Leu) (red, n = 11) with the mean and standard deviation, and circles indicate values from individual experiments. A two-tailed Mann-Whitney U-test demonstrated that the values were significantly different ($p = 0.0005$, Mann-Whitney U = 13). Source data are provided as a Source Data file.

Section 13. Characteristics of patients with protein truncating variants.

Of the patients with loss-of-function variants, there were 10 patients with a protein truncating variant. These patients had an older mean age of onset of 13.5 months [CI 7-62] and notably a large proportion were inherited, with one *de novo*, eight inherited and one unreported. Absence seizures were commonly reported at follow up and there were no cases of focal seizures reported (Fig. S15). Four of the patients were seizure free after treatment. The intellectual disability ranged from normal (one) to severe (three), with one mild and five moderate cases of intellectual disability (Fig. S16).

Supplementary Figure S15.

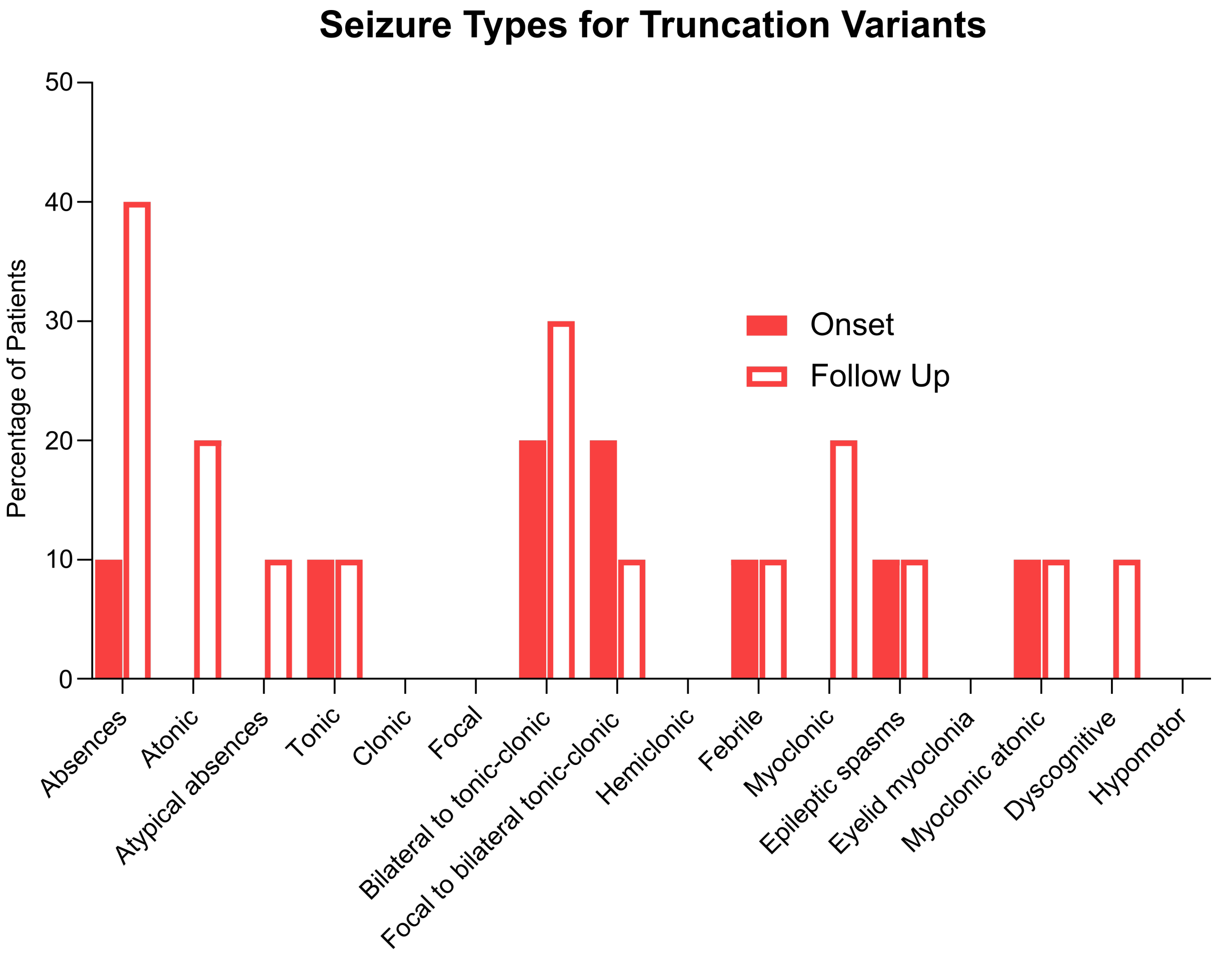

Seizure types of patients with protein truncating variants. Percentage of patients with variants of no functional change presenting with different seizure types at onset (filled bars) and follow-up (open bars).

Supplementary Figure S16

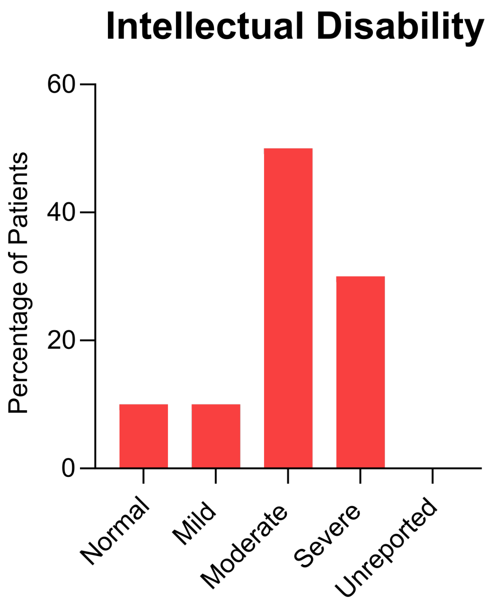

Intellectual disability of patients with protein truncating variants. Bar Graph of the percentage of patients with different severities of intellectual disabilities in patients with protein truncating variants.
