# Supplementary material for: Gain-of-function and loss-of-function GABRB3 variants lead to distinct clinical phenotypes in patients with developmental and epileptic encephalopathies
Source: Nat Commun. 2022 Apr 5;13:1822. doi: 10.1038/s41467-022-29280-x (PMC8983652; doi:10.1038/s41467-022-29280-x)
Supplement: Supplementary file 3 — Reporting Summary [file 41467_2022_29280_MOESM3_ESM.pdf]

nature portfolio

Corresponding author(s): Philip K Ahring

Last updated by author(s): Jan 6, 2022

Reporting Summary

Nature Portfolio wishes to improve the reproducibility of the work that we publish. This form provides structure for consistency and transparency in reporting. For further information on Nature Portfolio policies, see our Editorial Policies and the Editorial Policy Checklist.

Statistics

For all statistical analyses, confirm that the following items are present in the figure legend, table legend, main text, or Methods section.

n/a	Confirmed	
☐	☒	The exact sample size (*n*) for each experimental group/condition, given as a discrete number and unit of measurement
☐	☒	A statement on whether measurements were taken from distinct samples or whether the same sample was measured repeatedly
☐	☒	The statistical test(s) used AND whether they are one- or two-sided<br>*Only common tests should be described solely by name; describe more complex techniques in the Methods section.*
☐	☒	A description of all covariates tested
☐	☒	A description of any assumptions or corrections, such as tests of normality and adjustment for multiple comparisons
☐	☒	A full description of the statistical parameters including central tendency (e.g. means) or other basic estimates (e.g. regression coefficient) AND variation (e.g. standard deviation) or associated estimates of uncertainty (e.g. confidence intervals)
☐	☒	For null hypothesis testing, the test statistic (e.g. *F*, *t*, *r*) with confidence intervals, effect sizes, degrees of freedom and *P* value noted<br>*Give P values as exact values whenever suitable.*
☒	☐	For Bayesian analysis, information on the choice of priors and Markov chain Monte Carlo settings
☒	☐	For hierarchical and complex designs, identification of the appropriate level for tests and full reporting of outcomes
☒	☐	Estimates of effect sizes (e.g. Cohen's *d*, Pearson's *r*), indicating how they were calculated

Our web collection on statistics for biologists contains articles on many of the points above.

Software and code

Policy information about availability of computer code

Data collection: No software was used, clinical data were collected using a REDCap database that is institutionally licensed, as stated in the methods.

Data analysis: All data were analyzed with the commercial software programs pClamp 10.2, GraphPad Prism 8.2.1 and Spss 28.0 as stated in the methods.

For manuscripts utilizing custom algorithms or software that are central to the research but not yet described in published literature, software must be made available to editors and reviewers. We strongly encourage code deposition in a community repository (e.g. GitHub). See the Nature Portfolio guidelines for submitting code & software for further information.

Data

Policy information about availability of data

All manuscripts must include a data availability statement. This statement should provide the following information, where applicable:

- Accession codes, unique identifiers, or web links for publicly available datasets
- A description of any restrictions on data availability
- For clinical datasets or third party data, please ensure that the statement adheres to our policy

De-identified clinical data relevant for all statistics and conclusions in this manuscript has been provided in Supplementary Table S1. Further data in our *GABRB3* database that does not breach privacy will be made available to those eligible upon request. For functional data, the peak current amplitudes that were used to analyse $logEC_{50}$ values are provided in Supplementary Table S1. This also contains the original source filenames should requests be made for re-analysis of the data. Raw trace files will be stored for a minimum of seven years at the University of Sydney and are available to those eligible on request. *GABRB3* Transcript NM_000814.4/5 and pdb:6hup were used and gnomAD database was accessed for benign variants

Field-specific reporting

Please select the one below that is the best fit for your research. If you are not sure, read the appropriate sections before making your selection.

☒ Life sciences ☐ Behavioural & social sciences ☐ Ecological, evolutionary & environmental sciences

For a reference copy of the document with all sections, see nature.com/documents/nr-reporting-summary-flat.pdf

Life sciences study design

All studies must disclose on these points even when the disclosure is negative.

Sample size

As this is a rare disease, the sample size for clinical parameters were determined solely by the availability of patients with epilepsy and a known variant in the *GABRB3* gene.
For $logEC_{50}$ analysis, a power calculation was performed with the mean and standard deviation of a wild-type dataset, and a $logEC_{50}$ shift of 0.2 that was estimated from previous mutant analysis (e.g. Absalom et al, Brain Comm, 2021). A minimum sample size of 9 was determined, and we therefore rounded up to 10 for variants, and a minimum wild-type value of n(wt)=n(var)*sqrt(number of variants), as suggested for the Dunnett's post-hoc test (72). As wild-type experiments were run concurrently with variants, this value was exceeded.

A similar calculation for maximum currents with a change of 0.5 estimated a minimum sample size of 14. However, as a non-parametric test were used this value is not necessarily accurate, hence we increased the minimum value to 20.

Data exclusions

Clinical data were not excluded.

For electrophysiology experiments, data were excluded where the holding current were significantly altered during the experiment, the initial three applications of control did not yield consistent current or excessive run up or run down of currents from the same GABA concentration were observed.

Replication

A minimum of two batches of oocytes on two separate recording days were used for each experiment and similar numbers of experiments were taken from each run.
In several cases (E77K, T287I, Y302C), experiments were performed independently by two different researchers. In such case no differences were observed between data obtained in by the researchers

Randomization

Randomization was not relevant to the study, as there were no control or treatment groups to be assigned.

Blinding

Researchers performing the initial data analysis had access to the identity of each variant, but not the clinical information until experiments were complete.

Reporting for specific materials, systems and methods

We require information from authors about some types of materials, experimental systems and methods used in many studies. Here, indicate whether each material, system or method listed is relevant to your study. If you are not sure if a list item applies to your research, read the appropriate section before selecting a response.

Materials & experimental systems

n/a	Involved in the study
☒	☐ Antibodies
☒	☐ Eukaryotic cell lines
☒	☐ Palaeontology and archaeology
☐	☒ Animals and other organisms
☐	☒ Human research participants
☒	☐ Clinical data
☒	☐ Dual use research of concern

Methods

n/a	Involved in the study
☒	☐ ChIP-seq
☒	☐ Flow cytometry
☒	☐ MRI-based neuroimaging

Animals and other organisms

Policy information about studies involving animals; ARRIVE guidelines recommended for reporting animal research

Laboratory animals

Xenopus laevis females of 1-5 years of age were used solely to extract tissue (i.e. oocytes) - no animal experimentation was performed.

Wild animals

This study did not involve wild animals

Field-collected samples

This study did not involve field samples.

Ethics oversight	A protocol for Xenopus laevis extraction was approved by the Animal Ethics Committee of The University of Sydney (AEC No. 2016/970) in accordance with the National Health and Medical Research Council of Australia code for the care and use of animals.

Note that full information on the approval of the study protocol must also be provided in the manuscript.

Clinical data

Policy information about clinical studies

All manuscripts should comply with the ICMJE guidelines for publication of clinical research and a completed CONSORT checklist must be included with all submissions.

Clinical trial registration	The data was not part of a clincial trial.
Study protocol	The data was not collected as part of a clinical trial.
Data collection	This is a retrospective descriptive study and not part of a clinical trial.<br>For 74 patients, data were collected from previous publications. For the 11 patients with unpublished data, *GABRB3* variants were collected through collaborations with epilepsy and genetic centres in Europe and Canada in some cases facilitated via GeneMatcher. Clinical information was collected by face-to-face interviews with patients and their families and from clinical charts. Signed consent forms were obtained for all 11 patients.
Outcomes	Not relevant, since this is a retrospective descriptive study, and not part of a clinical trial
